# Cytochrome P450 isoforms 1A1, 1B1 AND 2W1 as targets for therapeutic intervention in head and neck cancer

**DOI:** 10.1038/s41598-021-98217-z

**Published:** 2021-09-23

**Authors:** Daniela Presa, Syed A. Khurram, Amir Z. A. Zubir, Sneha Smarakan, Patricia A. Cooper, Goreti R. Morais, Maria Sadiq, Mark Sutherland, Paul M. Loadman, James McCaul, Steven D. Shnyder, Laurence H. Patterson, Klaus Pors

**Affiliations:** 1 Institute of Cancer Therapeutics, School of Pharmacy and Medical Sciences, Faculty of Life Sciences, University of Bradford, Bradford, BD7 1DP West Yorkshire UK; 2 Unit of Oral and Maxillofacial Pathology, School of Clinical Dentistry, 19 Claremont Crescent, Sheffield, S10 2TA UK; 3 Present Address: Regional Maxillofacial Unit, Queen Elizabeth University Hospital, 1345 Govan Road, Glasgow, G51 4TF UK

**Keywords:** Cancer, Drug discovery

## Abstract

Epidemiological studies have shown that head and neck cancer (HNC) is a complex multistage process that in part involves exposure to a combination of carcinogens and the capacity of certain drug-metabolising enzymes including cytochrome P450 (CYP) to detoxify or activate such carcinogens. In this study, CYP1A1, CYP1B1 and CYP2W1 expression in HNC was correlated with potential as target for duocarmycin prodrug activation and selective therapy. In the HNC cell lines, elevated expression was shown at the gene level for CYP1A1 and CYP1B1 whereas CYP2W1 was hardly detected. However, CYP2W1 was expressed in FaDu and Detroit-562 xenografts and in a cohort of human HNC samples. Functional activity was measured in Fadu and Detroit-562 cells using P450-Glo™ assay. Antiproliferative results of duocarmycin prodrugs ICT2700 and ICT2706 revealed FaDu and Detroit-562 as the most sensitive HNC cell lines. Administration of ICT2700 in vivo using a single dose of ICT2700 (150 mg/kg) showed preferential inhibition of small tumour growth (mean size of 60 mm^3^) in mice bearing FaDu xenografts. Significantly, our findings suggest a potential targeted therapeutic approach to manage HNCs by exploiting intratumoural CYP expression for metabolic activation of duocarmycin-based prodrugs such as ICT2700.

## Introduction

Head and neck cancers (HNCs) are a heterogeneous group of cancers which can develop in several areas within the head and neck such as the oral cavity, nasopharynx, oropharynx, hypopharynx, larynx, paranasal sinuses and salivary glands^1^. These diseases are defined by different histological subtypes of which the most common is head and neck squamous cell carcinoma (HNSCC, SCC), which usually begins in the moist epithelial cell layers of the mucous membranes. Collectively, HNSCC is the sixth most common malignancy worldwide, accounting for over 90% of all HNC-related cancer^2^. Although inherited disorders such as Fanconi anaemia have been linked with predisposition to HNC due to genetic-related susceptibility, risk factors generally linked to HNC disease are strongly associated with lifestyle and environmental factors^3^. High rate of morbidity, potential for disfigurement, poor prognosis in the later stages and lack of timely detection makes HNC a difficult disease to address. Chemotherapeutic drugs approved for treating HNCs include platinating agents (carboplatin and cisplatin), antimetabolites (5-fluorouracil and capecitabine) and taxanes (docetaxel), which are intrinsically systemically toxic and are employed with varying degrees of success in terms of survival benefit^4^. Over the past couple of decades many advancements in cancer genetics have revealed new possibilities in both detection and treatment of HNC. The first molecularly targeted treatment in HNC was cetuximab which acts through binding of the epidermal growth factor receptor (EGFR). This monoclonal antibody has been found to be beneficial for HNC patients with locally advanced and recurrent tumour when administered along with routine chemotherapeutics or radiotherapy. More recently, nivolumab and pembrolizumab, two checkpoint inhibitors that target the programmed death-1 (PD-1) receptor, have also emerged as treatment options for both localised and metastatic HNCs^5,6^. To improve on survival and quality of life for HNC patients, further research is required to provide new insights into earlier detection and new selective treatments that target tumour-expressed proteins or pathways for therapeutic intervention.

Cytochrome P450 (CYP) enzymes play a major role in the biotransformation of exogenous (drugs, chemicals, natural products) and endogenous compounds (eicosanoids, liposoluble vitamins, steroids, bile acids) highlighting the catalytic versatility of this class of enzymes^7^. Even though the majority of CYP enzymes are predominantly expressed in the liver, elevated expression of CYP family members has been detected in HNC patients as well. CYP1A1, 1B1 and 2W1 have been shown to be expressed at significant levels in HNC patients, although CYP2W1 expression has been reported only at the mRNA level^8–10^. A number of meta-analyses have shown an association between CYP1A1 polymorphisms and HNC^11^. Accordingly, the individual susceptibilities to HNC have been correlated with two functional nonsynonymous polymorphisms, Ile462Val and MspI. These could alter CYP1A1 expression and function, potentially influencing the balance between metabolic activation and detoxification of toxicants^12^. It has also been shown that the inter-individual differences in CYP1A1 expression or function might contribute to the variability in risk towards various types of environmental exposure that influence this neoplasm^8^. One of the risk factors is smoking which, through the activation of tobacco carcinogens by CYP1A1, plays an essential part in the pathogenesis of HNC^11^. With regard to CYP1B1, immunohistochemical labelling of cell lines and tissue microarrays demonstrated significantly higher levels in SCC patients compared to controls^9^. CYP1B1 enzyme activity against procarcinogens and gonadal steroid hormones is influenced by polymorphisms such as Leu432Val and Asn453Ser^13^. A meta-analysis study suggests that the risk of SCC development, especially in Caucasians is significantly associated with the variation in Leu432Val^9^ while a recent study supported a role for polymorphic variants to drive cancer cell stemness and patient outcome in HNC^14^. CYP2W1 expression in HNC remains relatively understudied with the exception of a recent publication which showed that CYP2W1 mRNA expression is not related to betel-quid chewing but could be induced in smokers^10^. Despite some evidence of the CYP isoform not interacting with the classical CYP redox partners POR or b5^15^, capacity for metabolising a number of endogenous and exogenous compounds^16–22^ and for being under epigenetic control in e.g. colon tissues^23^, the role of CYP2W1 is poorly understood. Nonetheless, the clinical relevance of CYP2W1, either as a biomarker of malignancy and/or potential as drug target remains an exciting opportunity^15,24–26^.

As a continuation of our interest in investigating CYPs overexpressed in tumour tissue^26–30^ we show CYP1A1, 1B1 and 2W1 expression in HNC as potential targets for therapeutic intervention using CYP-activated duocarmycin bioprecursors developed in-house.

## Materials and methods

### Cell culture

The human HNC cell lines A-253, Detroit-562, FaDu, OSC19, SCC4, SCC5, SCC10, SCC14 and SCC16A were obtained from American Type Tissue Culture Collection (ATCC) (Manassas, VA, www.atcc.org). The isogenic colon cancer cell line pair SW480/ SW480 2W1, CHO/CHO 1A1 and the glioma cell line U87 were procured from Karolinska Institute, Stockholm, Sweden, University of Dundee, Dundee, Scotland and University of Turku, Finland respectively. Cells were grown as monolayer cultures in the respective medium (see Table S1 for detailed information) supplemented with 10% (v/v) FBS (Sigma), 1 mmol/L sodium pyruvate, and 2 mmol/L of l-Glutamine at 37 °C, 5% CO_2_ and 100% humidity until cells reached 75–85% confluence, at which point they were passaged using 25% trypsin/EDTA or progressed for experimental investigations. The cell lines were used between passage three and seven with all experiments performed at least in triplicates. All cell lines were authenticated morphologically and tested regularly for the absence of any mycoplasma infection. Cancer-associated fibroblast (CAF) (origin: floor of the mouth and lateral tongue) were kindly provided by Dr Helen Colley (South Sheffield Ethics Approval Committee Ref: 13/NS30120, STH17021)^31^. The conditioned media (CM) was prepared by culturing the fibroblasts with serum-free media (SFM; growth media without FCS) for 24 h, filtered (0.22 µM) and stored at − 20 °C.

### Real time polymerase chain reaction (RT-PCR)

The expression of CYP mRNA was quantified using RT-PCR assay in an Abi 7500 Real time PCR System (Applied Biosystems, Foster City, USA). The experiments were performed using with PrecisionPLUS MasterMix—Taqman-style, CYP1A1 (CAAGGTGTTAAGTGAGAAGGTG) and CYP1B1 (CACTGGAAACCGCACCTC) primers from Primerdesign® and CYP2W1 (GCATCCAGCCAGAGACAGG) primer from Applied Biosystems. Human (Primerdesign®) and hamster (TaqMan®) β-actin were used as endogenous controls. The gene expression was primarily determined relative to β-actin by applying the comparative ΔCt Method^32^. All samples were analysed in triplicate and by independent experiments. P values were determined using the two-tailed student t-test in Microsoft Excel 2013®; and the results were considered significant when P ≤ 0.05(*), P ≤ 0.01(**), P ≤ 0.001 (***).

### Immunohistochemistry

Immunohistochemistry (IHC) was performed on tissue sections from xenografts and frozen human samples. The latter were obtained from Ethical Tissue (http://www.bradford.ac.uk/business/ethical-tissue/), including participant consent for research purposes and approved by the University of Bradford’s ethical committee (application 15-067) to detect CYP1A1, 1B1 and 2W1 proteins. Paraffin-embedded tissues from human tumour xenografts were initially deparaffinised and dehydrated followed by antigen retrieval for 20 min which was performed by boiling the tissue sections in 10 mM citrate buffer (pH 6.0). After blocking, the sections were incubated in a humidified chamber overnight at 4 °C with the CYP1A1 (1:10), CYP1B1 (1:5) and CYP2W1 (1:1000) primary antibodies (see Table S1 for detailed information) diluted in the respective blocking reagents followed by incubation with MOM biotinylated anti-mouse IgG reagent. The slides were washed and incubated for another 30 min at RT with Avidin/Biotin Complex (VECTASTAIN ABC HRP Kit) and developed using 3, 3-diaminobenzidine (DAB) detection kit. The counterstaining was carried out using Harris’ haematoxylin. The tissue microarray (TMA) slides used to analyse the expression of CYP1A1, 1B1 and 2W1 were from the same tissue core (HN803c from Biomax) to minimise the difference between handling and preparation. The clinical characteristics are described in supplementary data. Quantification of immunohistochemical staining was carried out using specific IHC tools: slides were scanned to obtain digital whole slide images (WSI) using a Leica CS2 scanner, images were taken using Aperio Image scope® (Leica Biosystems, Version 12.4.2.5010) and quantification performed using Image J software (version 1.49, NIH, USA)^33^ with the IHC profiler macro add on^34^. Statistical analysis was carried out using GraphPad Prism 5.01 software, with Shapiro–Wilk normality test and one-way ANOVA (Dunnett's Multiple Comparison Test) used to determine statistical significance.

### Immunofluorescence staining

Cells seeded on coverslips were permeabilised using 0.1% Triton X-100 for 15 min at RT, followed by 1 h incubation with 5% BSA in order to block non-specific binding. After blocking, the cells were stained overnight at 4 °C for CYP1A1 (1:10), CYP1B1 (1:5) or CYP2W1 (1:1000) followed by incubation with the secondary antibodies Alexa Fluor 546 goat Anti-Mouse IgG and Alexa Fluor 488 goat Anti-Rabbit IgG for CYP1A1/1B1 and 2W1, respectively. DAPI was used as a counter stain. For analysis, the images were captured using a Leica DM 2000 fluorescence microscope.

### Chemical compounds

ICT2700, 2706 and 2726 have been reported before^26^ and used with a purity greater than 97%. A 10 mmol/L stock solution of the compound was prepared in DMSO.

### In vitro chemosensitivity assay

The MTT (3-(4,5-dimethyl-2-thiazolyl)-2,5-diphenyl-2H-tetrazolium bromide) assay was used to determine cell viability of HNC cell lines after exposure to ICT2700, 2706 and 2726^35^. The cell suspensions were prepared at cell density of 5 × 10^3^ cells/ml for FaDu, Detroit-562 and OSC19 and for all other cell lines at 1 × 10^4^ cells/ml and allowed to adhere overnight at 37 °C. A range of concentrations of the duocarmycin compounds were prepared (0.01–20 µM with 1% v/v DMSO) in medium and conditioned media harvested from primary oral CAFs. Plates were incubated at 37 °C in 5% CO_2_ incubator for 24, 48 and 72 h. 20 µL of 5 mg/mL MTT was added into each well of the microplate and incubated for 4 h at 37 °C. The solution of medium and MTT was removed and 150 μL of DMSO (Sigma) was added to dissolve the blue formazan crystals. Absorbance was recorded at 492 nm using a Tecan spectrometer and data analysed using Microsoft® Excel 2013 software. The IC_50_ was calculated from graphs with five dose–response points and at least three independent experiments for each compound per cell line. The formula used for the calculation is as follows: % survival = (absorbance of treated − absorbance of blank)/(absorbance of control − absorbance of blank) × 100.

### Antitumour activity

This investigation was conducted in accordance with ethical standards approved by the Animal Welfare Ethics Review Board at the University of Bradford, and in accordance with the UK National Cancer Research Institute Guidelines for the Welfare of Animals^36^. Throughout the study, all mice were housed in air-conditioned rooms in facilities approved by the United Kingdom Home Office to meet all current regulations and standards. All procedures were carried out under a Project Licence (PPL PFA0B4B35) issued by the UK Home Office according to government legislation and in compliance with ARRIVE guidelines (https://arriveguidelines.org/arrive-guidelines/). Female Balb/c immunodeficient nude mice (Envigo, Loughborough, U.K.), between the ages of 7 and 12 weeks were used. The mice were housed in cages in isolation cabinets with regular alternating cycles of light and darkness and received Teklad 2018 (Envigo) diet and water ad libitum. For the subcutaneous xenograft efficacy studies, mice were briefly given general anaesthesia and 2–3 mm^3^ fragments of FaDu tumour from donor xenografts were transplanted subcutaneously in the abdominal flanks of the efficacy study mice. Once all tumours were palpable, mice were randomised into treatment groups. Treated animals received a single 150 mg/kg dose of ICT2700, administered intraperitoneally on day 0, with control animals receiving solvent (10% DMSO: 90% arachis oil). Tumour volume, using callipers, and animal body weight were recorded throughout the experiments and normalised to the respective volume on the initial day of treatment (day 0). A Mann–Whitney *U*-test was used for analysis of significance in the first study, whilst a t-test was used to compare small (mean size of 60 mm^3^) and large (mean size of 240 mm^3^) tumours in the second study.

## Results

### Immunohistochemical expression in HNC frozen tissues and TMA

**Figure 1:**
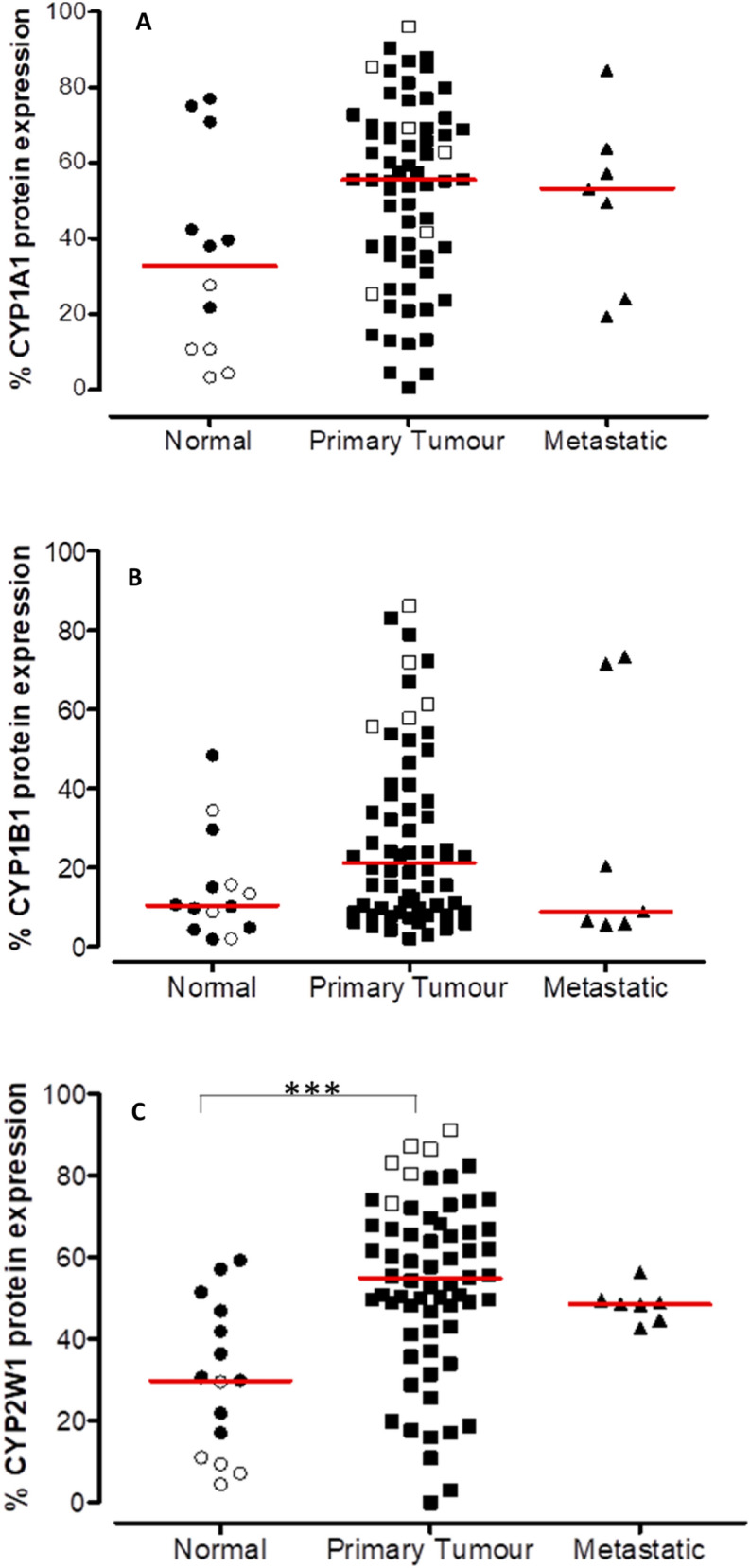
CYP1A1 (**A**), CYP1B1 (**B**) and CYP2W1 (**C**) protein expression in normal, primary tumour and metastatic patient samples. Black symbols represent TMA samples and white are from frozen tissue while the red line represents the median. ***P ≤ 0.001 relatively to normal tissue samples.

The expression of CYP1A1, 1B1 and 2W1 was measured in primary tumours from snap-frozen human HNC tissues and tissue microarray (TMA). In TMA, the expression of CYP1A1 was weak in normal tissues and moderate in primary and metastatic tumours (Fig. 1A). With CYP1B1, moderate expression was identified in 21% of the normal tissues, 34% of the primary tumours and 30% of the metastatic samples, with just 3% of tumours showing high expression of CYP1B1 (Fig. 1B). It is important to note that most of the normal samples from the frozen tissues were negative while the primary tumours in TMA exhibited moderate expression.

Expression of CYP2W1 in the normal tissues samples was found to be variable between TMA and frozen sections, barely detectable in the latter. However, the majority of tumour samples expressed CYP2W1 at a moderate (23%) to high (65%) level (Fig. 1C). In metastatic samples, the CYP2W1 expression was between 43 and 52%, revealing no significant difference from primary tumour tissue. Likewise, in the frozen tissue samples the majority of normal tissue (80%) displayed no CYP2W1 expression while the primary tumours from the same source consistently showed high expression (Fig. 1C). In general, the difference in CYP2W1 expression between normal tissue and primary tumours was highly significant (P ≤ 0.0002). Taken together the data indicated that CYP1A1, 1B1 and 2W1 isoforms were, in general, moderately or highly expressed in HNC compared with normal tissues. This supports the potential for CYP-activated prodrugs for therapeutic intervention in HNC following patient profiling for tumour CYP1A1, 1B1 or 2W1 expression.

### Expression levels of CYP1A1, 1B1 and 2W1 genes in HNC

**Figure 2:**
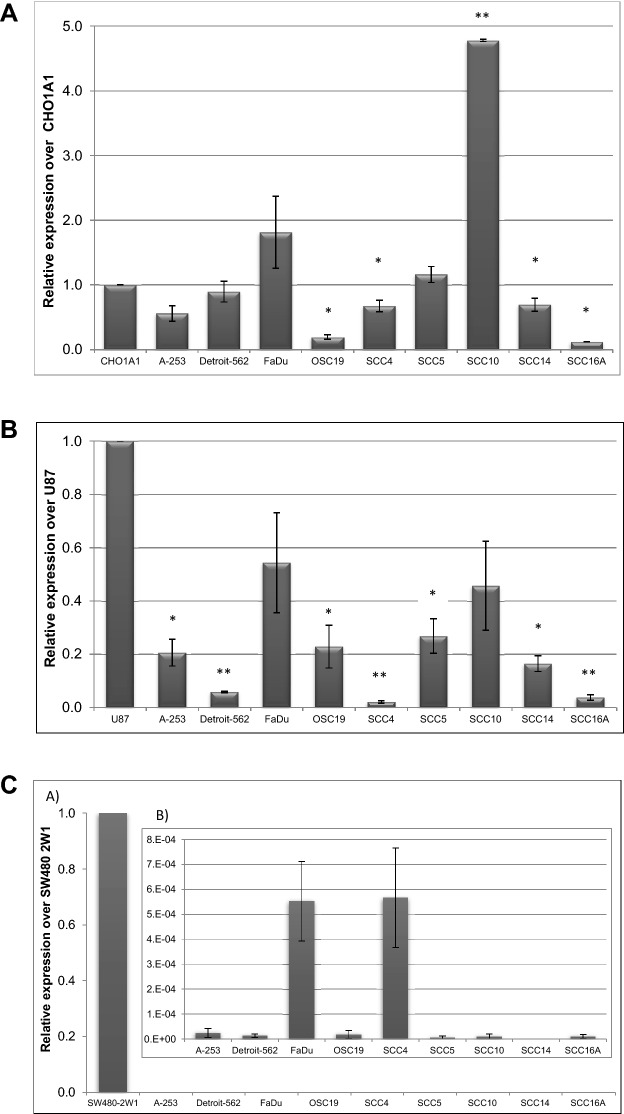
(**A**) Relative mRNA expression of CYP1A1 in a panel of HNC cell lines. Fold change relative to CHO1A1. Values are the mean of 3 independent experiments and error bars represent standard deviation (SD). [*P ≤ 0.05; **P ≤ 0.01 relatively to CHO1A1]. (**B**) Relative mRNA expression of CYP1B1 in a panel of HNC cell lines. Fold change relative to U87. Values are the mean of 3 independent experiments and error bars represent standard deviation (SD). [Relatively to U87: *P ≤ 0.05; **P ≤ 0.01]. (**C**) Relative mRNA expression of CYP2W1 in a panel of HNC cell lines. Fold change relative to SW480 2W1. Relative expression over CYP2W1 with scale bar between 0 and 1 (**A**) and scale bar between 0 to 8 × 10^–8^ (**B**). Values are the mean of 3 independent experiments and error bars represent standard deviation (SD).

Given the elevated expression of target CYPs in clinical samples we next went on to profile a panel of HNC cell lines and xenografts. CYP1A1, 1B1 and 2W1 at transcriptional and translational level was performed to explore their prevalence in HNCs. Accordingly, RT-PCR was carried out in HNC cell lines (n = 9) (A-253, Detroit-562, FaDu, OSC19, SCC4, SCC5, SCC10, SCC14 and SCC16A) using specific primers to determine the relative gene level expression of the CYPs. Isogenic cell line pairs CHO/CHO1A1^37^, SW480/SW480-2W1^26^ and the glioma cell line U87^38^ were used as controls for the expression of CYP1A1, 2W1 and 1B1, respectively. Evaluation of the HNC cell lines revealed varying levels of CYP1A1 expression in cells (Fig. 2A). FaDu, SCC5 and SCC10 were shown to have higher CYP1A1 expression although significant statistical difference (P ≤ 0.01) with the CHO1A1 cell line was observed only with SCC10. Contrarily, cell lines OSC19, SCC4, SCC14 and SCC16A had significantly lower expression (P ≤ 0.05) compared to CHO1A1. As opposed to CYP1A1, expression of CYP1B1 and CYP2W1 in HNC cell lines was found to be significantly low compared to the control cells. Reduced expression of CYP1B1 was observed in all cell lines except for the moderate expression in FaDu and SCC10 cell lines (Fig. 2B) while the expression of CYP2W1 in the majority of the cell lines examined was either low or absent (Fig. 2C). The results indicate that CYP1A1 and CYP1B1 have elevated mRNA level expression in HNC cell lines when compared to the CYP2W1 isoform. To corroborate these findings, we performed an in silico analysis of the RNA data from Cancer Cell Line Encyclopaedia (CCLE) dataset for HNC cell lines using the public cBioPortal website^39^ and found that the isoforms CYP1B1 and CYP2W1 were amplified in Detroit-562 and SCC4 respectively (Fig. S1).

### Immunodetection of CYP1A1, 1B1 and 2W1 in HNC

To understand if the mRNA expression levels correlated with protein, immunodetection assays were performed on cell lines, cell lined-derived xenografts (CDXs) and frozen tumour tissues. A summary of CYP1A1, 1B1 and 2W1 gene and protein expression can be found in Table 1.

**Table 1:**
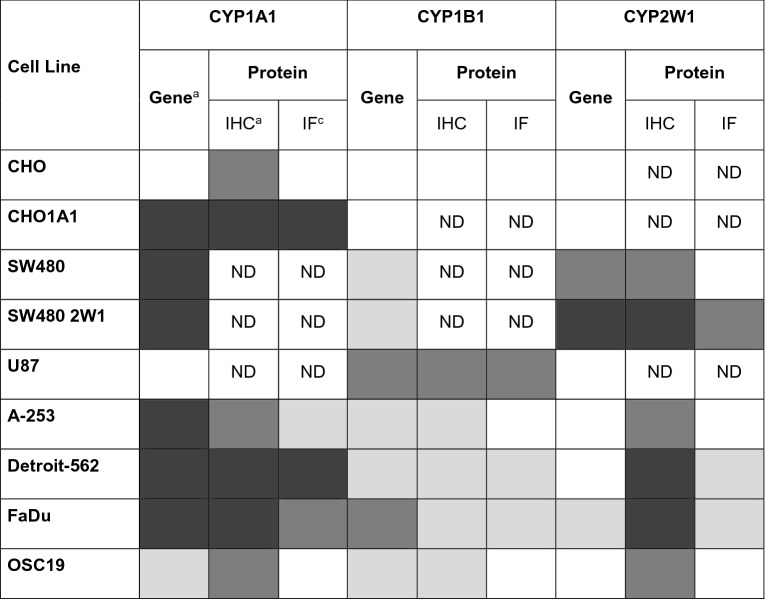
Summary of CYP1A1, 1B1 and 2W1 gene and protein expression levels.

^a^RT-PCR expression in Fig. 2A–C, ^b^expression by immunofluorescence in Fig. S2A–C, ^c^expression by immunohistochemistry in Figs. 3 and S3A–C; *ND* not determined. White—no expression, light gray—low expression, gray—moderate expression, dark gray—high expression.

#### Immunofluorescence of CYP1A1, CYP1B1 and CYP2W1 in HNC cell lines

CYP1A1 was detected in A-253, Detroit-562, FaDu and CHO1A1 (positive control) by immunofluorescence but not CHO and OSC19 cell lines (Fig. S2A). CYP1B1 expression was detected in Detroit-562 and FaDu cells along with the U87 glioma cells (positive control^38^), but absent in A-253 and OSC19 (Fig. S2B). It is notable that the expression of CYP1B1 in the cytoplasmic regions of Detroit-562 and FaDu were at a much lower level in comparison to U87. On the other hand, CYP2W1 displayed variable levels of expression in A253, Detroit-562, FaDu and OSC19 cells and CYP2W1-transfected SW480 colon cancer cells used as a positive control (Fig. S2C).

#### Immunohistochemical expression in HNC cell line derived xenografts (CDXs)

**Figure 3:**
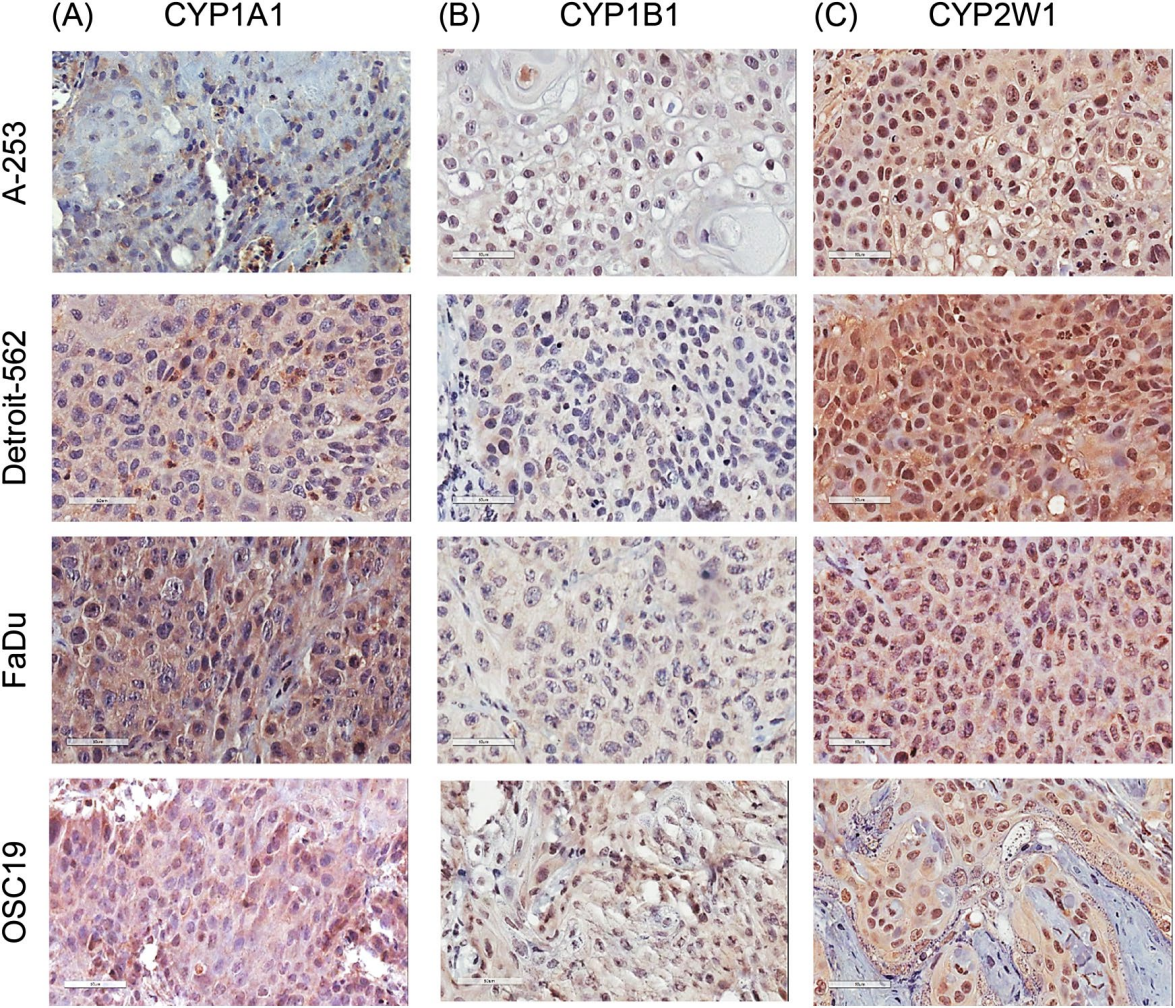
Immunohistochemical staining of CYP1A1 (**A**), CYP1B1 (**B**) and CYP2W1 (**C**) in tumour xenografts. Scale bar = 50 μm at 40 × magnification.

Expression of CYP1A1, 1B1 and 2W1 were further evaluated in a panel of selected xenograft tissues consisting of A-253, Detroit-562, FaDu and OSC19 cells as well as in primary human HNC sections by immunohistochemistry. Highest expression of CYP1A1 was observed in Detroit-562 and FaDu with expression being extra-nuclear (Fig. 3A). In comparison to CHO, FaDu (P ≤ 0.001), CHO1A1 (P ≤ 0.001) and Detroit-562 (P ≤ 0.01) showed significantly higher expression of CYP1A1 (Fig. S3A).

CYP1B1 expression was observed to be weak in A-253, Detroit-562, FaDu and OSC19 (Fig. 3B). The expression of CYP1B1 in all examined cell lines were mainly identified in the cytoplasm. In general, the expression was significant between cell lines (P ≤ 0.001) and also in comparison with CHO, U87 and OSC19 was significant (P ≤ 0.001, Fig. S3B). The expression of CYP2W1 was shown to be high in Detroit-562 and FaDu and SW480-2W1, and moderate in A-253, SW480 and OSC19 cells. The expression of CYP2W1 was predominantly cytoplasmic, with the intensity being proportional to the presence of CYP2W1 protein (Fig. 3C). Compared to SW480 (Fig. S3C), CYP2W1 expression was significantly higher in SW480-2W1 (P ≤ 0.001), Detroit-562 (P ≤ 0.001), FaDu (P ≤ 0.001) and A253 (P ≤ 0.01).

### Growth inhibition of duocarmycin compounds in HNC cell lines

Next, we investigated three duocarmycin-based compounds ICT2700, ICT2706 and ICT2726 previously explored for CYP-activation; the former two duocarmycins are bioactivated by CYP1A1 and 2W1 to potent cytotoxins^25–27^ while the latter analogue is inactivate^37^ and hence here employed as a negative control when evaluated against a panel of HNC cell lines. Detroit-562 cells were the most sensitive to ICT2700 (IC_50_ = 280 nM) followed by Fadu, SCC5, SCC10 and SCC16A cell lines (IC_50_ circa 1 µM). ICT2706 was not as potent as ICT2700, which is consistent with previous data in CYP1A1-expressing cells^25,26^. Antiproliferative activity was not observed for ICT2726 under the conditions investigation (IC_50_ ≥ 10 µM, Table 2) consistent with previous observations, hence the inclusion here as a negative control compound^26^. Antiproliferative activity of ICT2700 and ICT2706 was confirmed using isogenic cell lines expressing target CYPs (CHO/CHO1A1 and SW480/SW480-2W1) as previously reported^26,27^.

**Table 2:** Growth inhibition of duocarmycin compounds in HNC cell lines.


Cell line	IC_*50*_ (µM)		
	ICT2700	ICT2706	ICT2726
A-253	2.3 ± 0.3	4.5 ± 0.9	> 25^a^
Detroit -562	0.28 ± 0.08	1.7 ± 0.1	9.8 ± 1.4
FaDu	1.2 ± 0.2	0.9 ± 0.2	< 25^a^
OSC19	5.1 ± 0.8	9.1 ± 0.0	< 25^a^
SCC4	11.6 ± 0.9	10.3 ± 0.2	< 25^a^
SCC5	0.9 ± 0.1	10.2 ± 2.2	> 10
SCC10	1.5 ± 0.4	> 10	> 10
SCC14	5.84 ± 0.1	10.9 ± 0.9	< 25^a^
SCC16A	1.02 ± 0.25	7.5 ± 0.6	< 25^a^

^a^Not soluble above this concentration.

**Figure 4:**
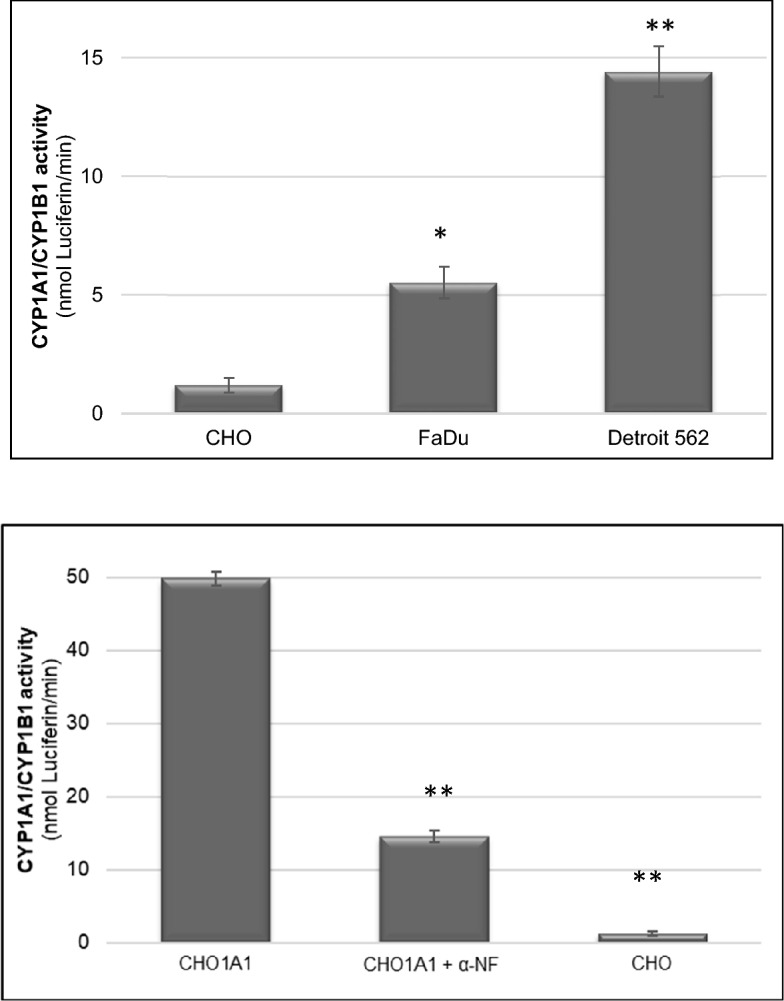
**(A)** CYP1A1/CYP1B1 activity in CHO, and the HNC cell lines FaDu and Detroit-562. [***P ≤ 0.001, **P ≤ 0.01 relatively to CHO cell line]. (**B**) CYP1A1/CYP1B1 activity in CHO1A1 and CHO cell lines in the absence or presence of 1 µM of the inhibitor α-NF and CHO. [***P ≤ 0.001 relatively to CHO1A1].

The anti-proliferative activity of the compounds observed in Fadu and Detroit-562 cells linked to functional CYP1A1 activity using a P450-Glo™ assay, which measures the luminescence produced via CYP1A1 mediated conversion of the substrate proluciferin to D-luciferin. An increase in CYP1A1 functional activity was observed in both FaDu and Detroit-562 cells although this was not as high as in CHO1A1-transfected cells (Fig. 4A); activity was reduced in the latter by treating cells with α-naphthoflavone, further indicating CYP1A1 activity (Fig. 4B). ICT2700 was observed to be most potent compound in HNC cell lines and hence was investigated in vivo.

### Effect of ICT2700 on tumour growth in FaDu xenografts

**Figure 5:**
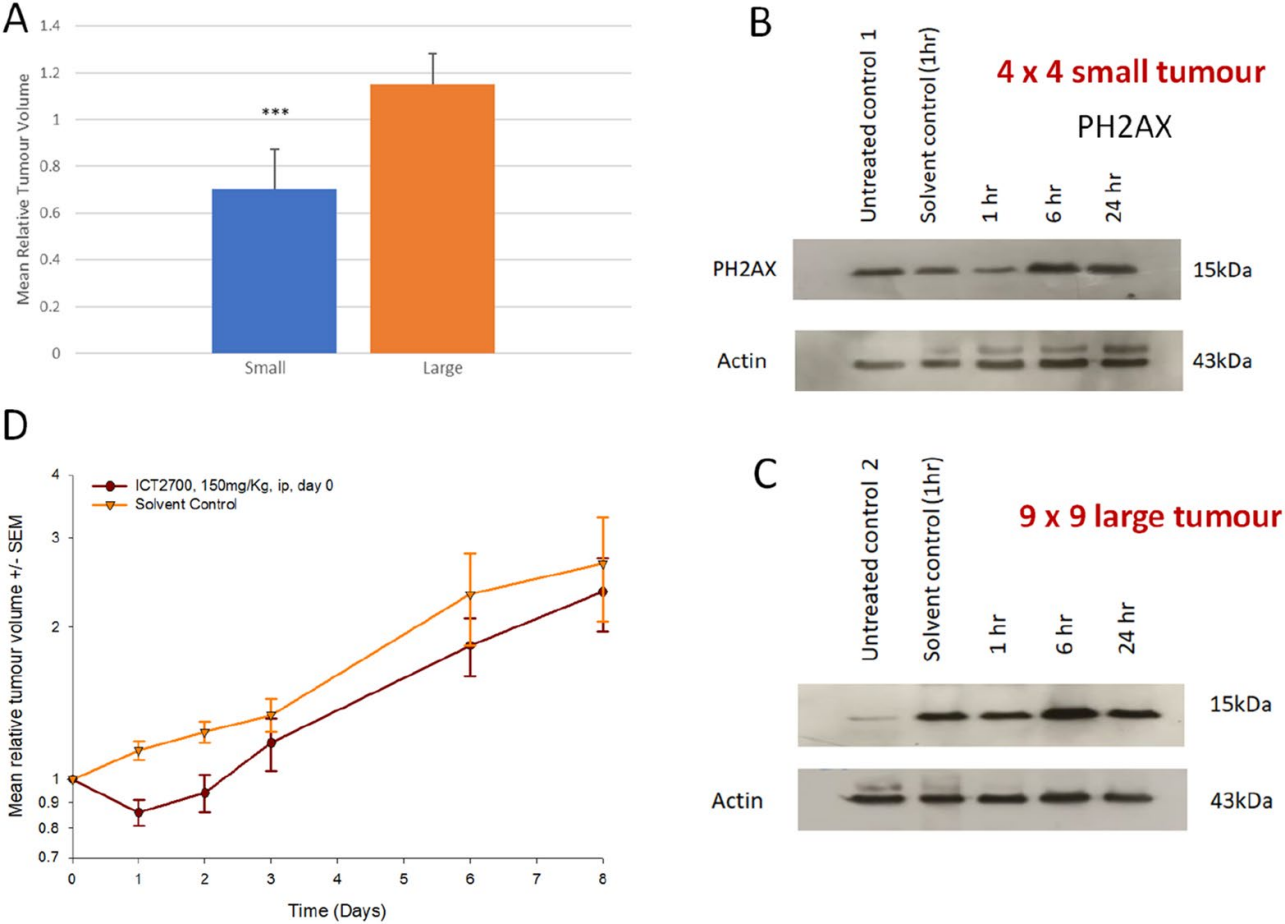
Administration of ICT2700 to FaDu tumours. Mice were treated with a single 150 mg/kg dose of ICT2700, i.p. either when tumours where a mean size of 60 mm^3^, or a mean size of 240 mm^3^ (**A**). γH2AX was used as a marker of DNA double strand damage after 1, 6 and 24 h time points in small (**B**) and large (**C**) tumours (full blots provided in Fig. S6). ICT2700 impact on tumour growth delay (**D**).

Whilst the Detroit-562 cell line exhibited a tenfold higher sensitivity to ICT2700 compared to other HNC cell lines in vitro, it had an inconsistent tumour take rate in vivo, and thus was unsuitable for use in efficacy studies. Instead, FaDu was selected for in vivo studies, as in vitro analysis indicated expression of CYP1A1, 1B1 and 2W1 isoforms and functional CYP1A1 activity of the former. Initially, we carried out a pilot study with FaDu tumours where mice were treated with a single 150 mg/kg dose of ICT2700, i.p. either when tumours where a mean size of 60 mm^3^, or a mean size of 240 mm^3^. Tumours were measured after 48 h and the relative tumour volume at day 2 were compared to the initial tumour volume. A significant (P = 0.0004) difference was seen in response to ICT2700 in tumours of a relatively small (mean size of 60 mm^3^) size at the commencement of treatment compared with the larger (mean size of 240 mm^3^) tumour cohort (Fig. 5A). Increased DNA damage was observed after 6 h compared with both 1 and 24 h time points (Fig. 5B-C) indicating generation of duocarmycin chemotoxin metabolite previously identified^27^ and responsible for cell death and reduction in tumour size. Next, ICT2700 was administered to mice bearing palpable tumours as a single dose on day 0 (150 mg/kg) and the impact on tumour growth delay and systemic toxicity (weight loss) was measured. Although an initial treatment effect was observed with negligible weight loss (Fig. S5), no statistically significant growth delay was observed after eight days (Fig. 5D).

### Effect of CAF derived conditioned media on the activity of ICT2700

**Figure 6:**
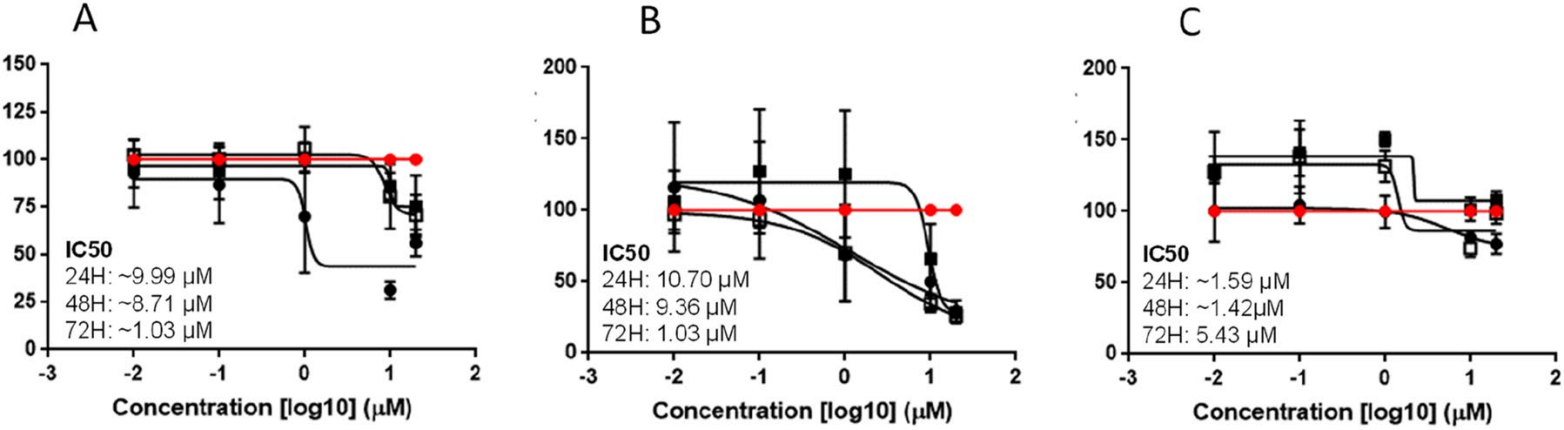
(**A–C**) Dose–response curve of ICT2700 treatment of FaDu-RPMI (**A**), FaDu-CAF002 (**B**) and FaDu-CAF003 (**C**) conditioned media viability for time-point 24, 48 and 72 h using Alamar Blue viability assay. Vehicle control was DMSO at 1% (v/v).

The tumour microenvironment (TME) is a complex multicellular system hosting a variety of immune and non-immune cell types and include cancer-associated fibroblasts (CAFs) as one of the most dominant components in the TME^40^. The TME contributes to tumourigenesis through a multifactorial process and some emerging evidence suggests differential expression of CYP1 enzymes in fibroblasts which might alter treatment sensitivity^41^. In accordance with this and to inform the in vivo efficacy of ICT2700, the effect of this prodrug in a co-culture of FaDu cells and HNC CAFs was investigated. Cells grown in the presence of conditioned media from cancer-associated fibroblast (CM-CAF) obtained from two HNC patients (CAF002 and CAF003) showed no change in the potency of ICT2700 (IC_50_: 1.03 µM) when compared with control Fadu cells grown in RPMI media alone (IC_50_: 1.03 µM). Moreover, FaDu cells co-cultured with CAF003 showed a time-dependent decrease in sensitivity to ICT2700. Specifically, the inhibition of cell growth by ICT2700 for FaDu/CAF003 co-culture (at 24 h) was IC_50_ of 1.59 µM in contrast to CAF002 (IC_50_: 10.07 µM) (Fig. 6).

## Discussion

Despite the multi-modality therapies used for the clinical management of HNCs involving surgery, radiotherapy, chemotherapy and targeted therapy the survival rates have not been improved significantly over the past few decades. Although progress has been made in biologic targeted therapies, notably cetuximab, no new targeted small molecules have been approved for clinical use. CYP superfamily isoforms responsible for drug oxidations are attractive as targets to selectively activate prodrugs in tumours^16,42–48^. The differential expression of selected CYPs in tumour versus surrounding normal tissue offers an excellent opportunity to develop targeted therapies with significantly reduced side effects. In the present study we have profiled CYP1A1, 1B1 and 2W1 expression in HNC and explored if these can be exploited for therapeutic gain. The mRNA and protein expression profiles of the three CYP isoforms were investigated in a panel of HNC cell lines, xenografts and primary tissues. Our results showed that the expression of CYP1A1/1B1 and 2W1 were poorly expressed in cell lines compared to the clinical and xenografts tissues, indicating in vivo microenvironment is important for CYP expression. This is further supported by an earlier study that was focused on the NCI 60 cancer cell line screen, and which revealed low CYP expression and functional activity in these immortalised cell lines^49^.

The FaDu cell line which expressed CYP1A1 and low levels of 2W1 as well as exhibiting functional CYP1A1 activity was used to investigate ICT2700 in vivo. ICT2700 delayed tumour growth but only in small sized tumours. γH2AX phosphorylation indicative of DNA damage was increased 6 h after treatment in both small and large tumours and correlates with bioprecursor bioactivation as previously shown^26,27^. All animals displayed normal appearance and behaviour with no significant loss in body weight throughout the course of these studies. The data supports that duocarmycin prodrugs typified by ICT2700 can be administered safely with good tolerability.

Emerging preclinical studies reveal the intriguing role played by several physical and biological factors of the TME in conferring intrinsic drug resistance to tumour cells^50^. Among the biological components, CAFs remain the most studied and predominant cell type of the TME which have been shown to enhance tumour growth and confer chemotherapy (e.g. cisplatin) resistance through an irreversible CAF-activation process and subsequent secretion of several growth factors and chemokines^51^. However, our in vitro analyses suggested that the co-culture of HNC tumour cells with conditioned medium obtained from culturing human CAFs from HNC patients did not impair the efficacy of ICT2700. This finding, while preliminary, suggests minimal influence of the TME on the potency of the CYP-targeted agents. In contrast, cisplatin, a drug used clinically to treat SSC, exhibited impaired cytotoxic activity in cells cultured with CM-CAFs (Fig. 6), which is in accordance with other studies that have shown CAFs to enhance drug resistance^52^.

CYP1A1 is a controversial target given that smokers induce this enzyme in the lung, but careful patient recruitment of only non-smokers could mean cohorts of HNC patients could benefit from CYP1A1-targeted therapeutics. CYP1B1 is highly expressed in many cancer types and has been known as a potential target for a long time^53,54^. Recent evidence suggests that never smokers make up an increasing proportion of the head and neck cancer population and now represent 24% of new cancer diagnoses (CRUK LIHNCS trial, McCaul et al. unpublished data). However very little progress has been made in exploiting this isoform for prodrug activation although there is much support from the development of CYP1B1 inhibitors^36^. In conclusion, this study indicates the potential of CYP expression as targets for therapeutic intervention in HNC and warrants further investigation of CYP function in HNC tumours and the TME. In addition, the dosing schedule of ICT2700 should be optimised to confirm the potential of this duocarmycin prodrug approach for HNC treatment.

## Supplementary Information

Supplementary Information.
