# Supplementary material for: Cytochrome P450 isoforms 1A1, 1B1 AND 2W1 as targets for therapeutic intervention in head and neck cancer
Source: Sci Rep. 2021 Sep 23;11:18930. doi: 10.1038/s41598-021-98217-z (PMC8460628; doi:10.1038/s41598-021-98217-z)
Supplement: Supplementary file 1 — Supplementary Information. [file 41598_2021_98217_MOESM1_ESM.docx]

**SUPPORTING INFORMATION**

**Cytochrome P450 isoforms 1A1, 1B1 AND 2W1 as targets for therapeutic intervention in head and neck cancer**

Daniela Presa^a^, Syed A. Khurram^b^, Amir Z. A. Zubir^b^, Sneha Smarakan^a^, Patricia A. Cooper^a^, Goreti R. Morais^a^, Maria Sadiq^a^, Mark Sutherland^a^, Paul M. Loadman^a^, James McCaul^a, c^, Steven D. Shnyder^a^, Laurence H. Patterson^a^, Klaus Pors^a*^

**Author Affiliations:**

^a^Institute of Cancer Therapeutics, School of Pharmacy and Medical Sciences, Faculty of Life Sciences, University of Bradford, Bradford BD7 1DP, UK.

^b^Unit of Oral and Maxillofacial Pathology, School of Clinical Dentistry, 19 Claremont Crescent, Sheffield S10 2TA, UK.

^c^Current address: Regional Maxillofacial Unit, Queen Elizabeth University Hospital, 1345 Govan Road, Glasgow, G51 4TF

***Correspondence:**

Dr. Klaus Pors,

Institute of Cancer Therapeutics,

Faculty of Life Sciences, University of Bradford,

West Yorkshire, BD7 1DP, UK.

E-mail: [k.pors1@bradford.ac.uk](mailto:k.pors1@bradford.ac.uk)

**Table S1.** Cell lines and growth medium requirements.

| **Cell lines** | **Tissue** | **Tissue source** | **Disease** | **Origin** | **Medium** |
| --- | --- | --- | --- | --- | --- |
| A-253 | salivary gland | primary | Epidermoid carcinoma | ATCC | McCoy’s 5A with 10% FBS and 1.5 mM L- glutamine (L-Gln). |
| CHO-DHFR | hamster ovary | primary | Normal | Dr.T Friedburg, University of Dundee, Dundee, Scotland | DMEM high glucose with 10% FBS, 2 mM L-Gln, 1% HT supplement |
| CHO1A1 |  |  |  |  | DMEM high glucose with 10% FBS, 2 mM L- Gln, 30 nM Methotrexate |
| Detroit-562 | pharynx | metastasis | Pharyngeal carcinoma | ATCC | MEME with 10% FBS, 2 mM L-Gln, 1 mM sodium pyruvate and 1% non-essential amino acids. |
| FaDu | pharynx | primary | SCC | ATCC |  |
| HEK293 Mock | Embryonic kidney | primary | Normal | Karolinska Institutet, Stockholm, Sweden | RPMI-1640 with 10% FBS, 2 mM L-Gln, 1 mM sodium pyruvate and 75 ng/ml Hygromycin B |
| HEK293 2W1 |  |  |  |  |  |
| OSC19 | tongue | metastasis | SCC | M.D. Anderson | DMEM high glucose with 10% FBS, 2 mM L- Gln, 1 mM sodium pyruvate |
| SCC4 | tongue | primary | SCC | ATCC | DMEM:F12 supplemented with 10% FBS, 2.5 mM L-Gln, 0.5 mM sodium pyruvate and 400 ng/ml hydrocortisone. |
| SW480 Mock | colon | primary | Colorectal Adenocarcinoma | Karolinska Institutet, Stockholm, Sweden | RPMI-1640 with 10%FBS, 2 mM L-Gln, 1 mM sodium pyruvate and 75 ng/ml Hygromycin B |
| SW480 2W1 |  |  |  |  |  |

**Table S1.** Cell lines and growth medium requirements - *continuation*.

| UT-SCC5 | tongue | primary | SCC | University of Turku,  Finland. | DMEM high glucose with 10% FBS, 2 mM L-Gln, 1 mM sodium pyruvate and 1% non-essential amino acids. |
| --- | --- | --- | --- | --- | --- |
| UT-SCC10 | tongue | primary |  |  |  |
| UT-SCC14 | tongue | primary |  |  |  |
| UT-SCC16a | tongue | primary |  |  |  |
| U87-MG | brain | primary | Glioblastoma | ATCC | RPMI-1640 supplemented with 10% FBS, 2 mM L-Gln, 1 mM sodium pyruvate. |

**Table S2.** Primary antibodies used

| **Primary antibody** | **Host species** | **Human amino acids** | **Manufacturers** |
| --- | --- | --- | --- |
| CYP1A1 | monoclonal mouse | PSLNAFKDLNEKFYSFMQKMVKEHYKTFEKGHIRDITDSLIEHCQEKQLDENANVQLSDEKIINIVLDLF | Santa Cruz Biotechnology  (sc-25304) |
| CYP1B1 | monoclonal mouse | PENFDPARFLDKDGL | a kind gift from the Prof Graeme Murray, University of Aberdeen |
| CYP2W1 | polyclonal rabbit | TMRPRAQALCAVPRP | a kind gift from the Prof Magnus Ingelman-Sundberg Karolinska Institutet, Stockholm, Sweden |


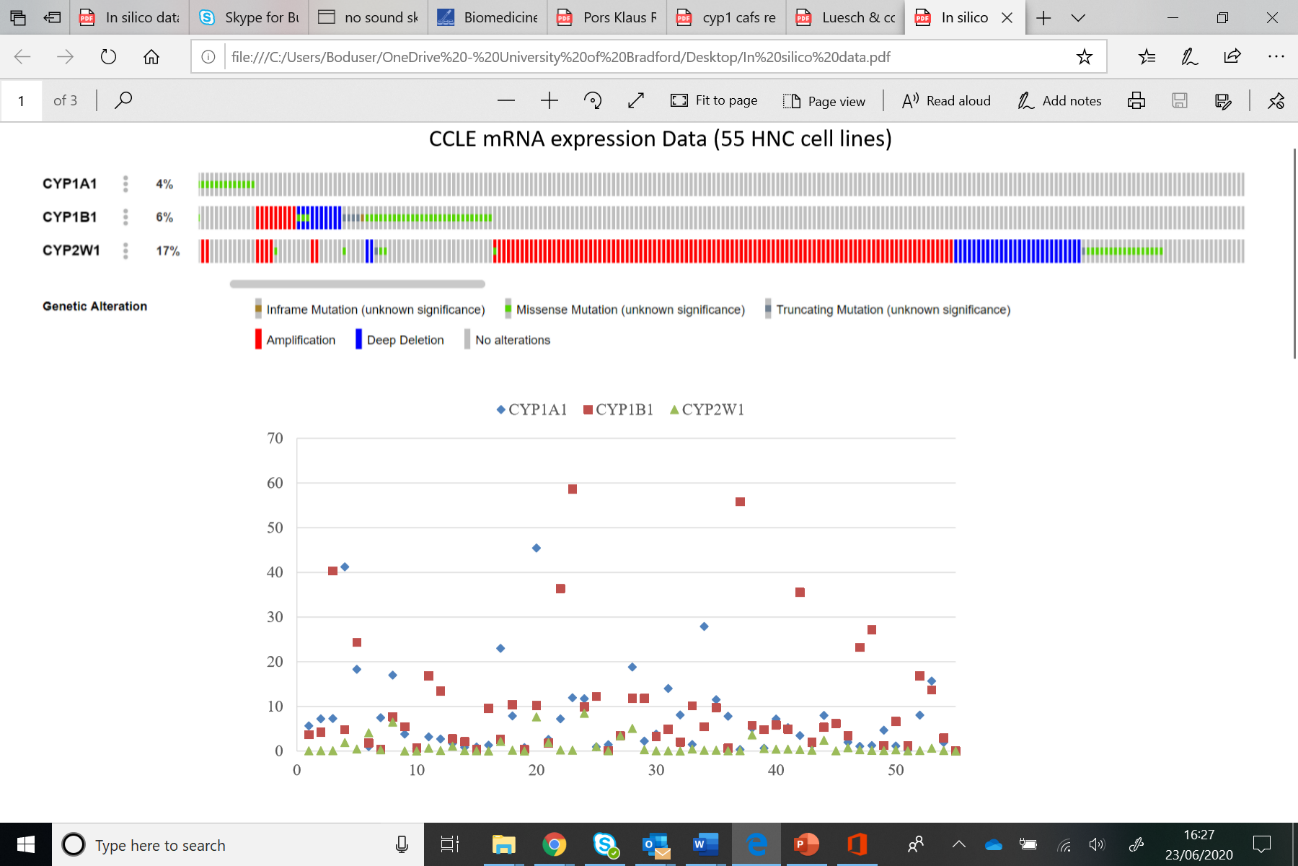


**Figure S1**. CCLE mRNA expression data (55 HNC cell lines)

|  | Dapi |  | CYP1A1 |  | Overlay |
| --- | --- | --- | --- | --- | --- |
| CHO | 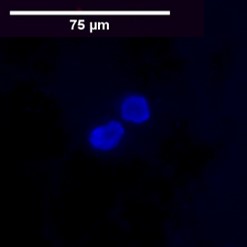 |  | 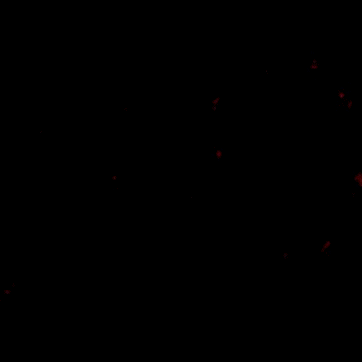 |  | 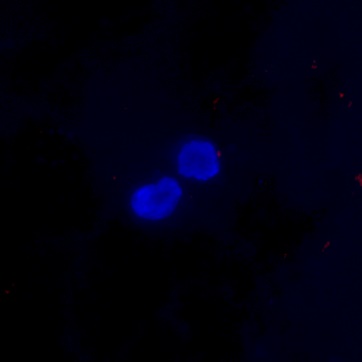 |
| CHO1A1 | 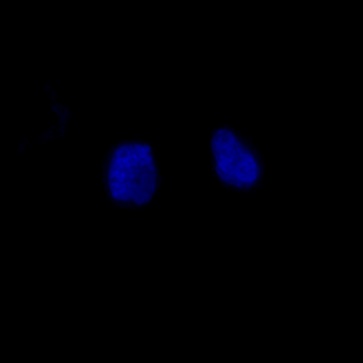 |  | 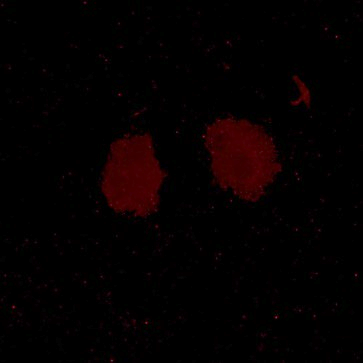 |  | 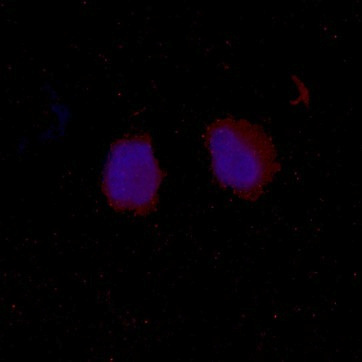 |
| A-253 | 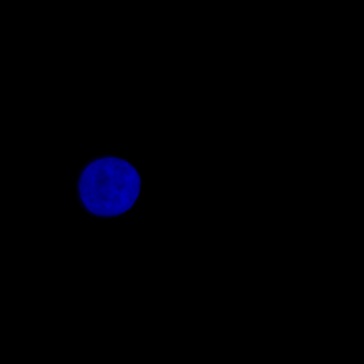 |  | 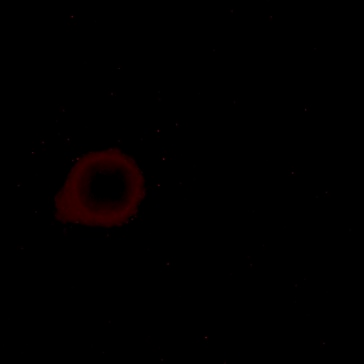 |  | 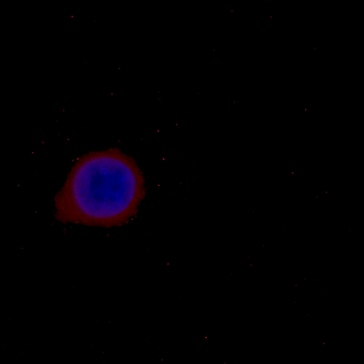 |
| Detroit-562 | 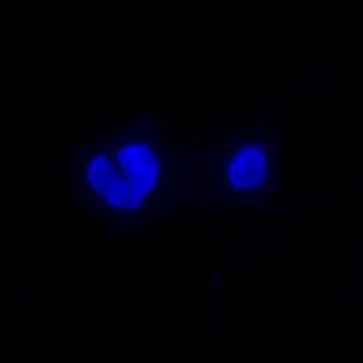 |  | 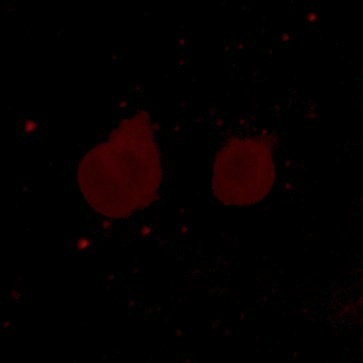 |  | 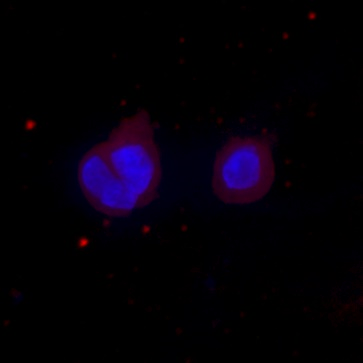 |
| FaDu | 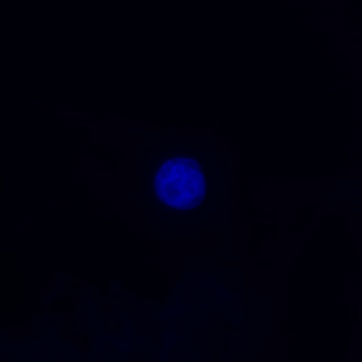 |  | 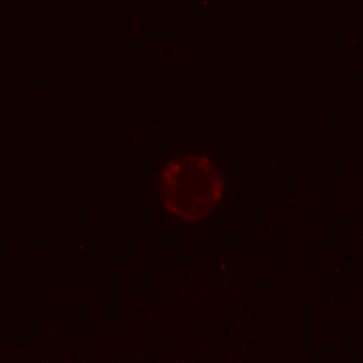 |  | 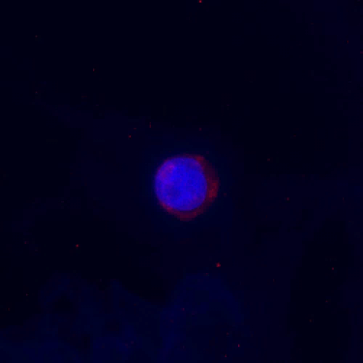 |
| OSC19 | 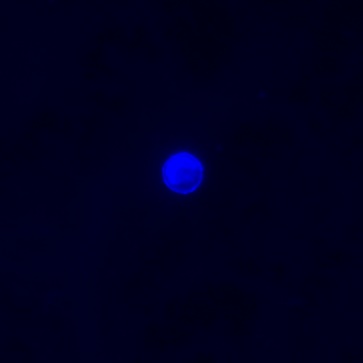 |  | 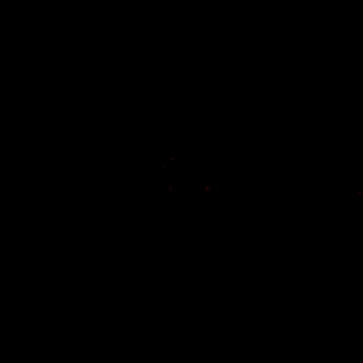 |  | 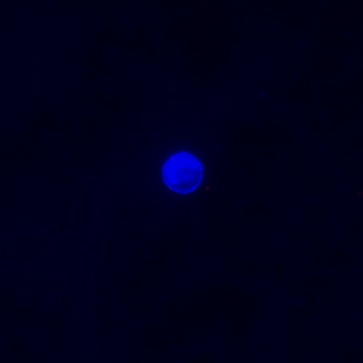 |

**Figure S2A**. Expression and subcellular location of CYP1A1 in cell lines. Blue label: nucleus; red label: CYP1A1. Scale bar = 75 μm.

|  | Dapi |  | CYP1B1 |  | Overlay |
| --- | --- | --- | --- | --- | --- |
| U87 | 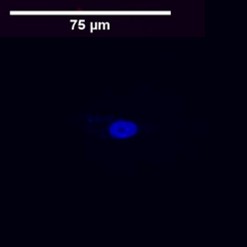 |  | 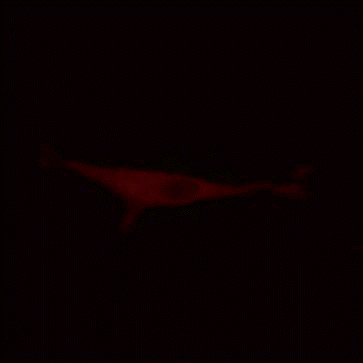 |  | 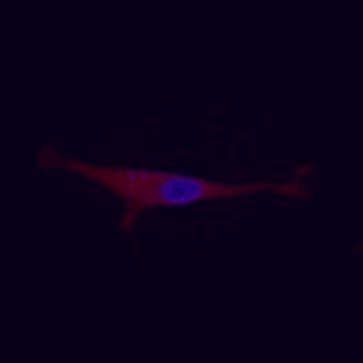 |
| A-253 | 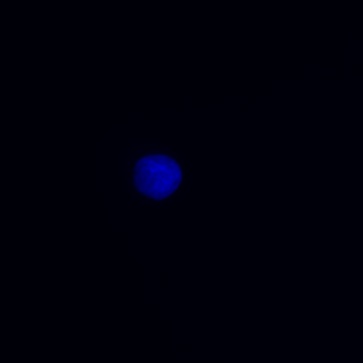 |  | 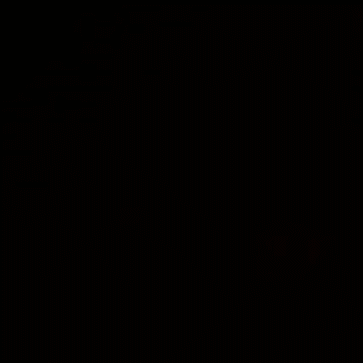 |  | 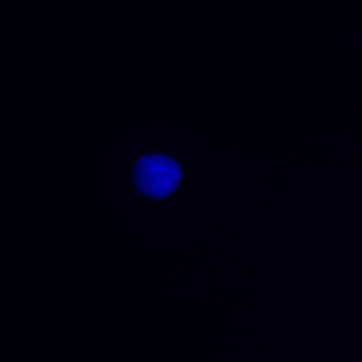 |
| Detroit-562 | 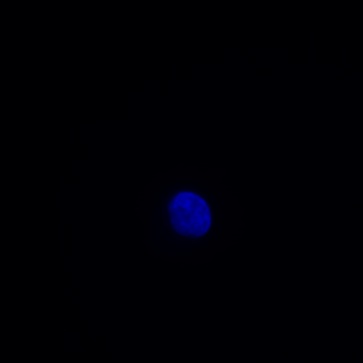 |  | 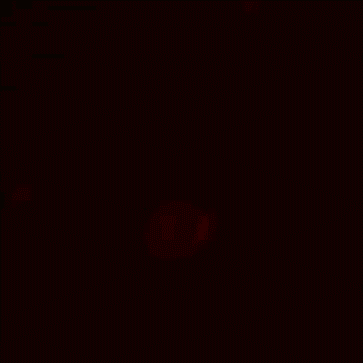 |  | 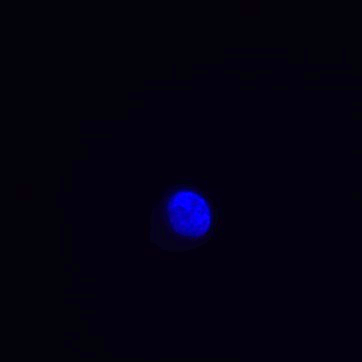 |
| FaDu | 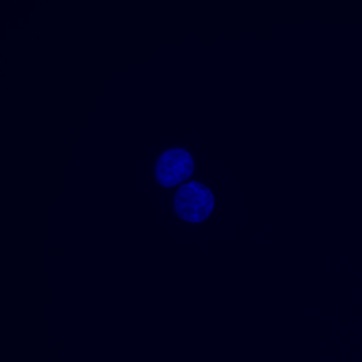 |  | 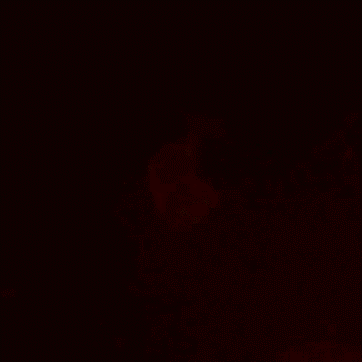 |  | 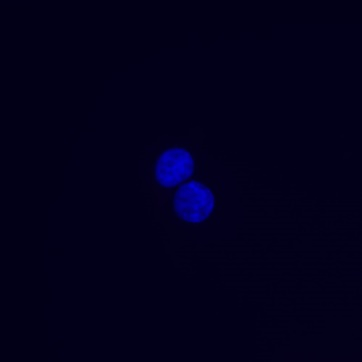 |
| OSC19 | 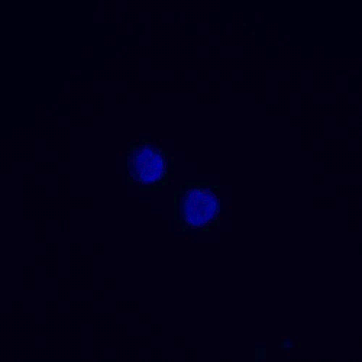 |  | 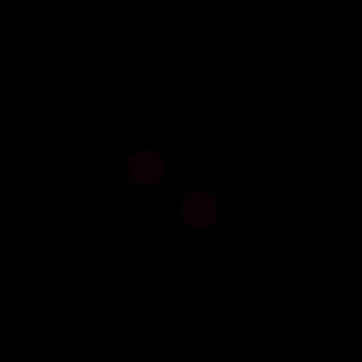 |  | 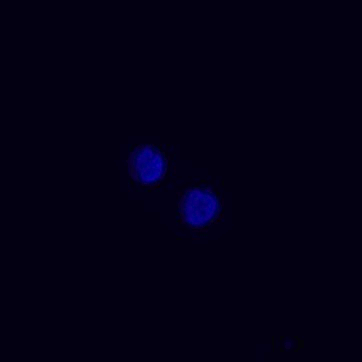 |

**Figure S2B.** Expression and subcellular location of CYP1B1 in cell lines. Blue label: nucleus; red label: CYP1B1. Scale bar = 75 μm.

|  | Dapi |  | CYP2W1 |  | Overlay |
| --- | --- | --- | --- | --- | --- |
| SW480 | 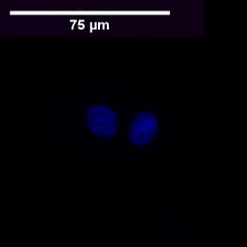 |  | 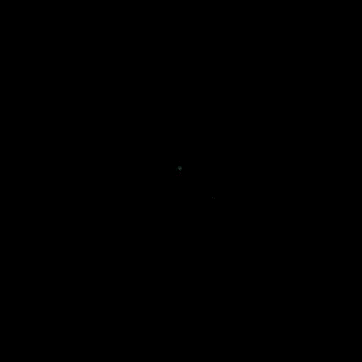 |  | 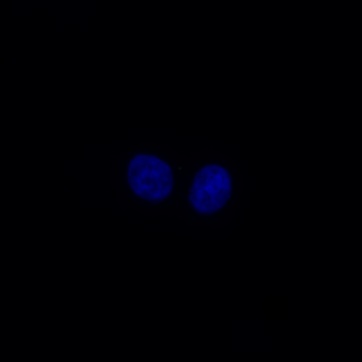 |
| SW480 2W1 | 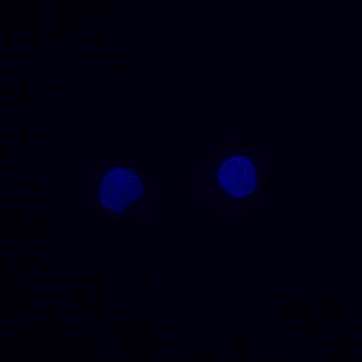 |  | 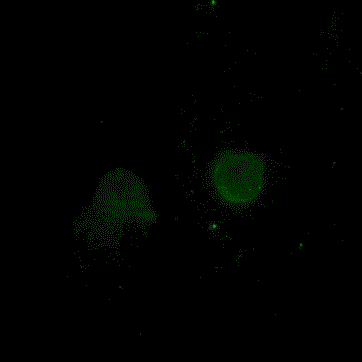 |  | 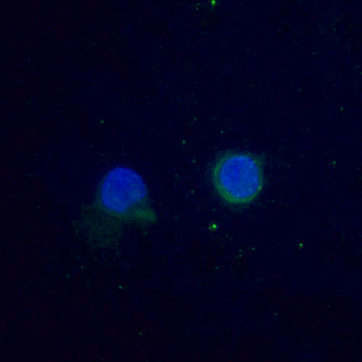 |
| A-253 | 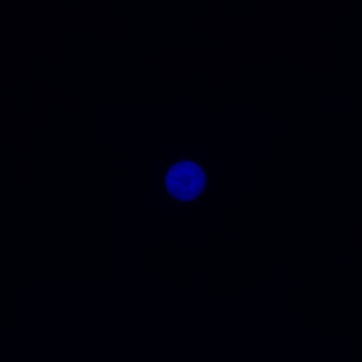 |  | 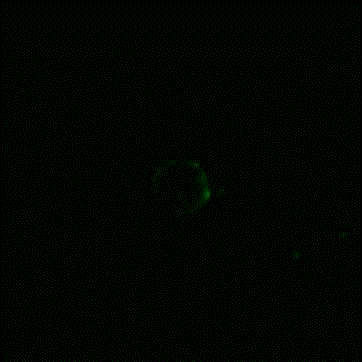 |  | 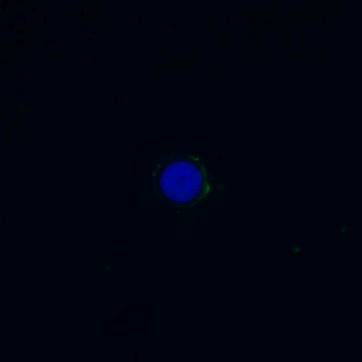 |
| Detroit-562 | 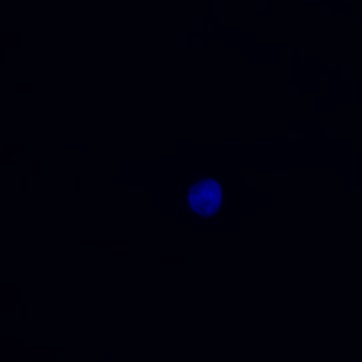 |  | 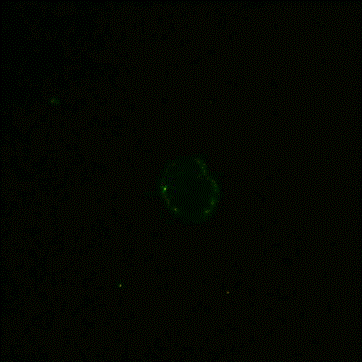 |  | 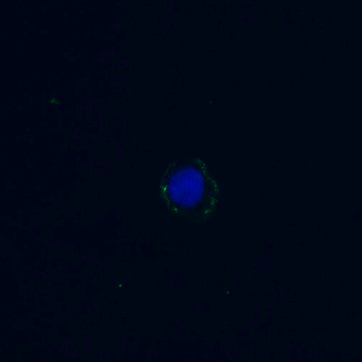 |
| FaDu | 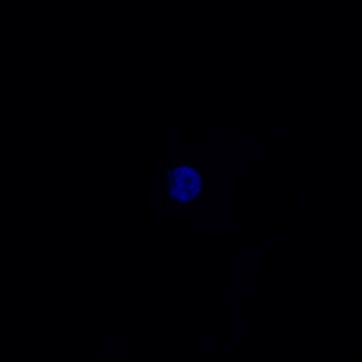 |  | 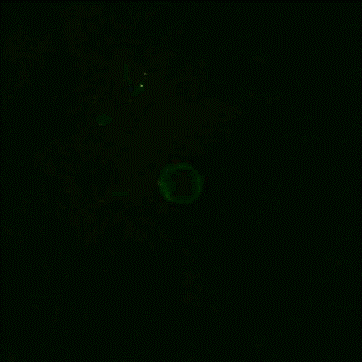 |  | 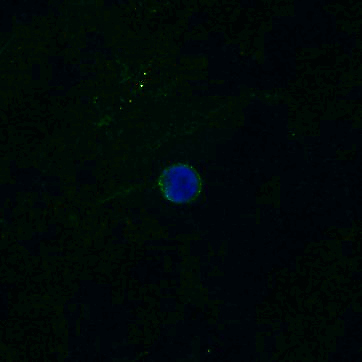 |
| OSC19 | 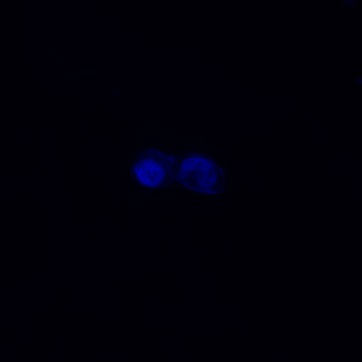 |  | 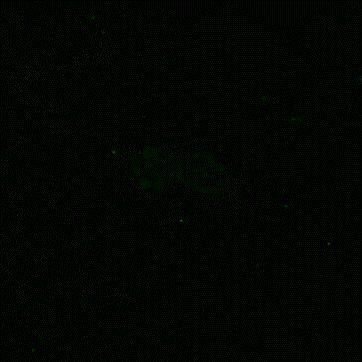 |  | 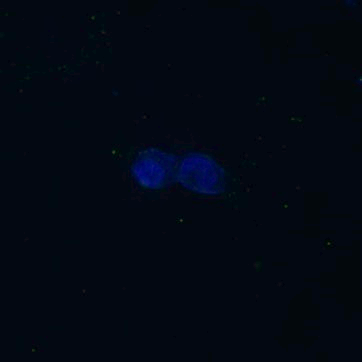 |

**Figure S2C.** Expression and subcellular location of CYP2W1 in cell lines. Blue label: nucleus; green label: CYP2W1. Scale bar = 75 μm.

|  | CYP1A1 |  | -ve control |
| --- | --- | --- | --- |
| CHO | 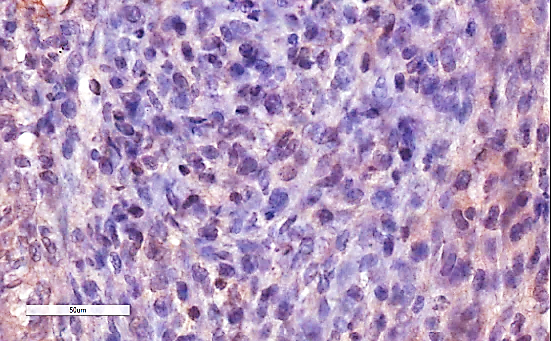 |  | 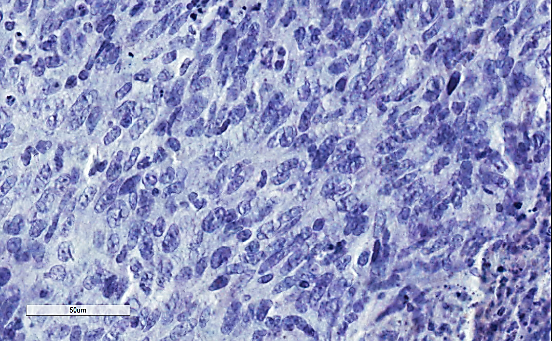 |
| CHO1A1 | 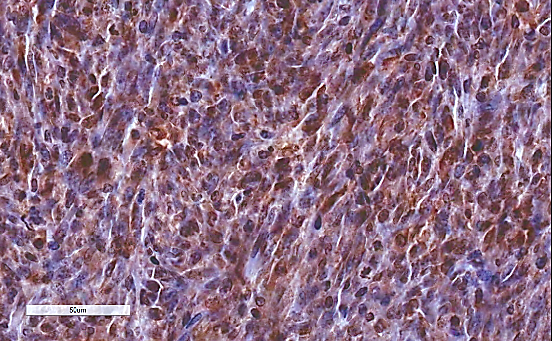 |  | 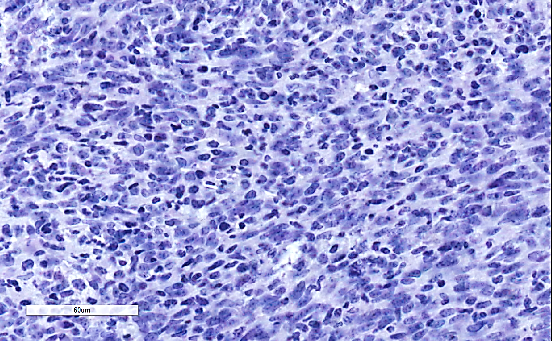 |
| A-253 | 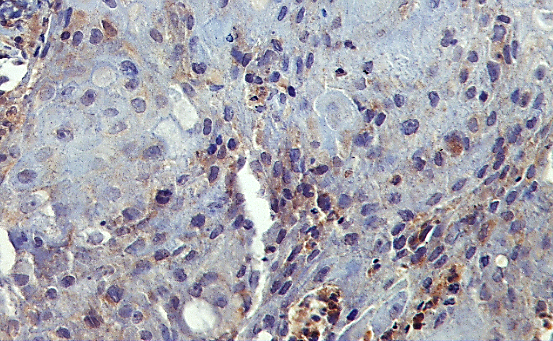 |  | 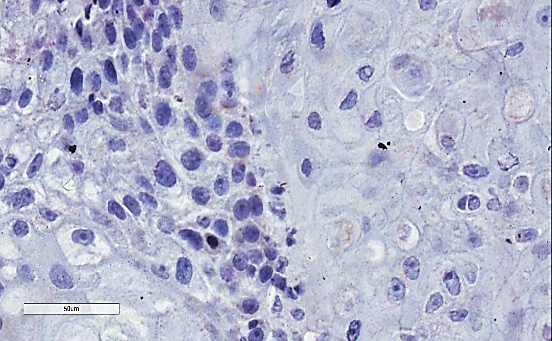 |
| Detroit-562 | 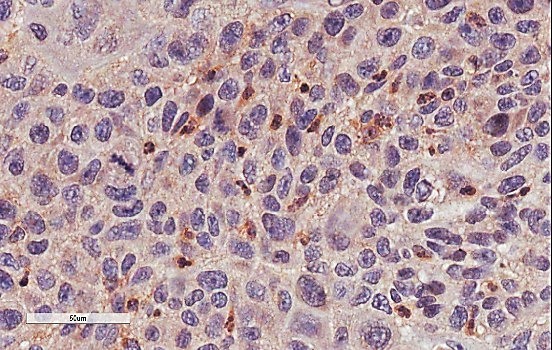 |  | 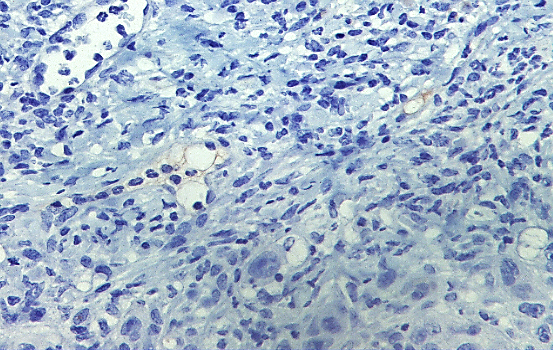 |
| FaDu | 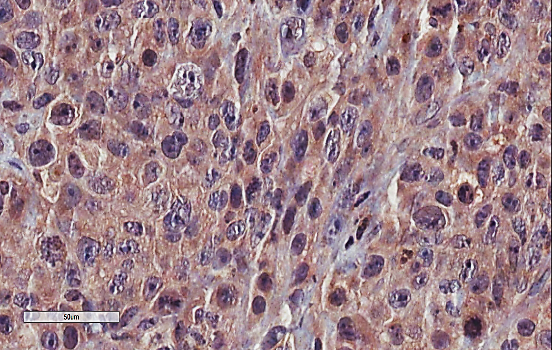 |  | 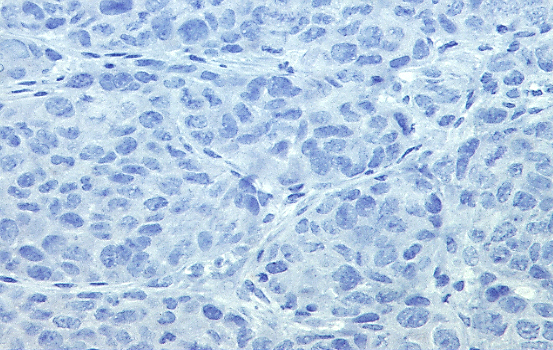 |
| OSC19 | 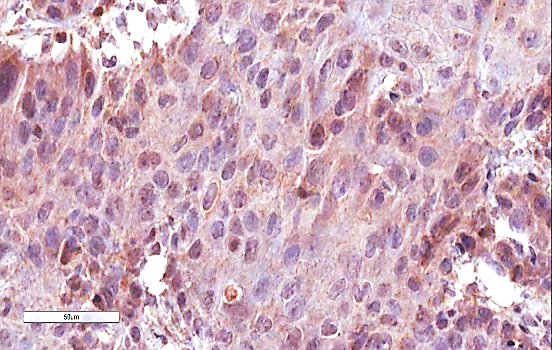 |  | 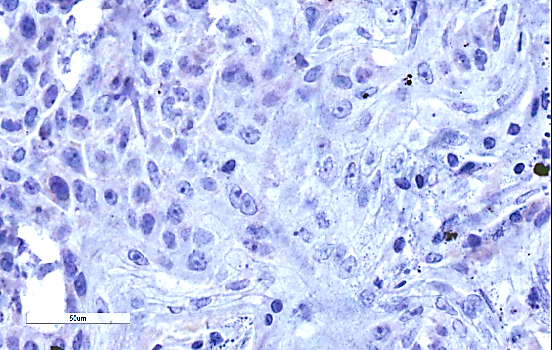 |

**Figure S3A.** Immunohistochemical staining of CYP1A1 in tumour xenografts and respective negative control (-ve control). Scale bar = 50 μm at 40x magnification.

|  | CYP1B1 |  | -ve control |
| --- | --- | --- | --- |
| CHO | 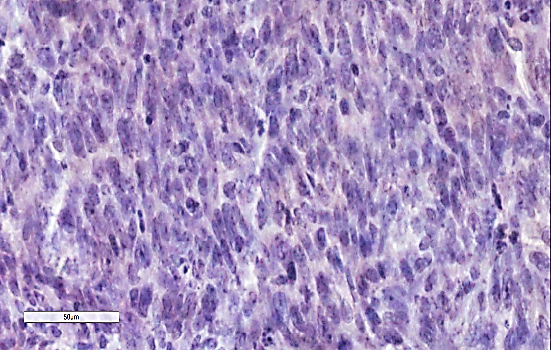 |  | 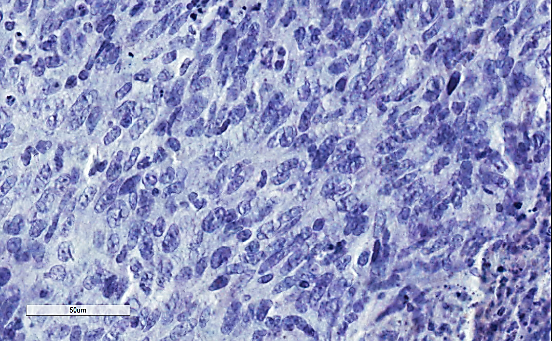 |
| U87 | 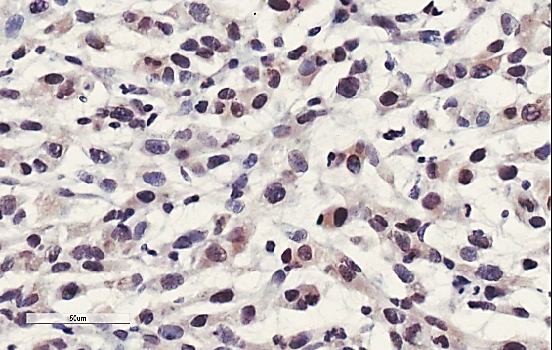 |  | 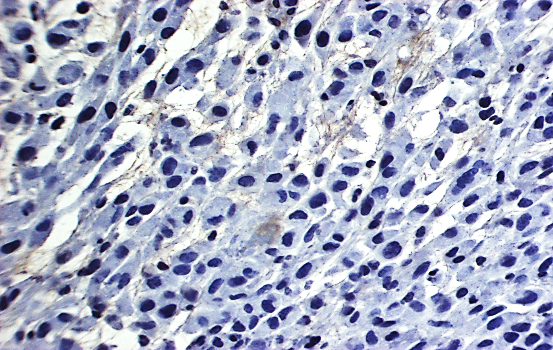 |
| A-253 | 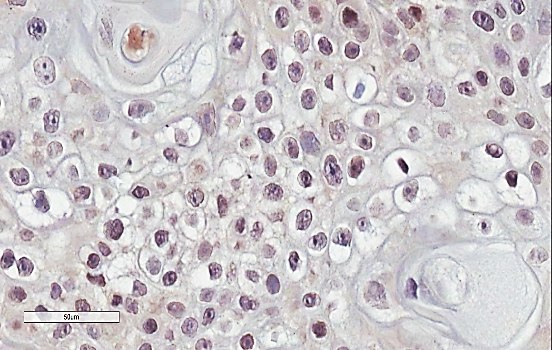 |  | 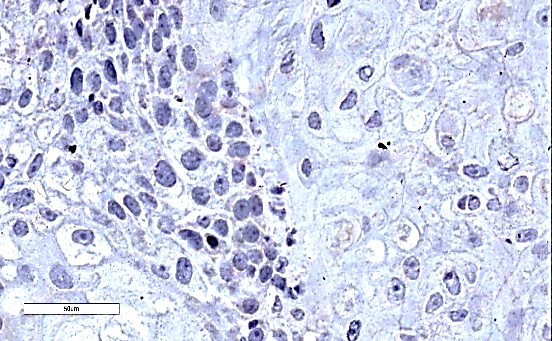 |
| Detroit-562 | 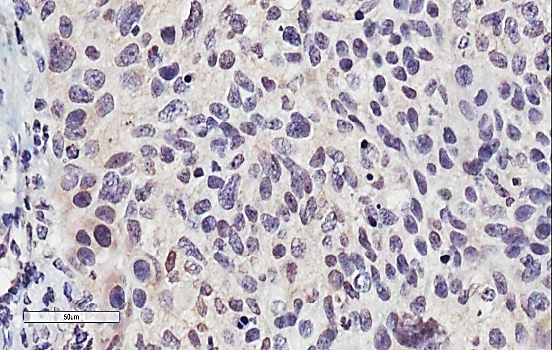 |  | 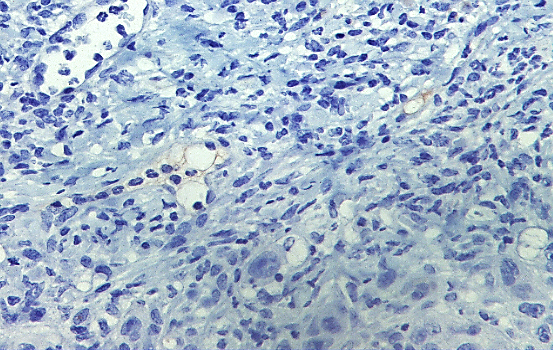 |
| FaDu | 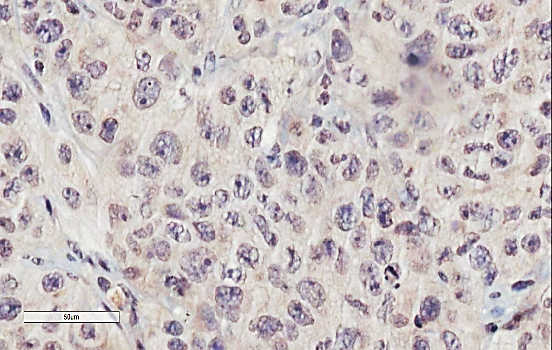 |  | 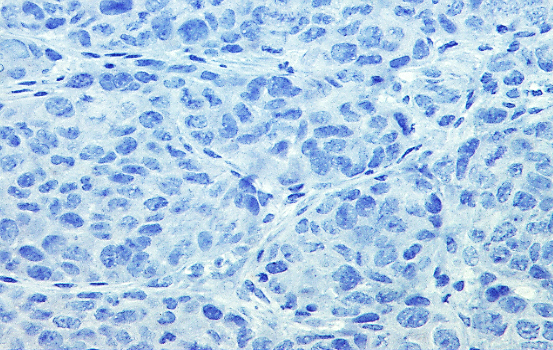 |
| OSC19 | 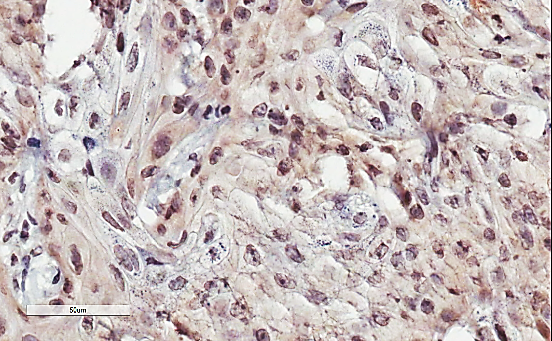 |  | 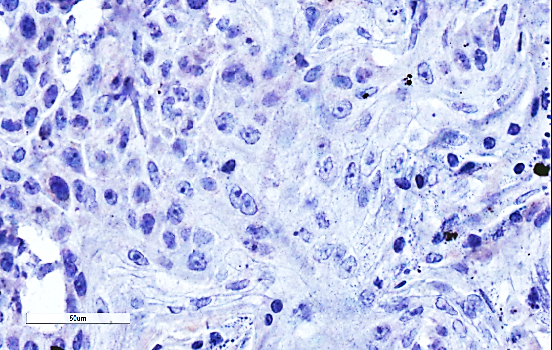 |

**Figure S3B.** Immunohistochemical staining of CYP1B1 in tumour xenografts. Scale bar = 50 μm at 40x magnification.

|  | CYP2W1 |  | -ve control |
| --- | --- | --- | --- |
| SW480 | 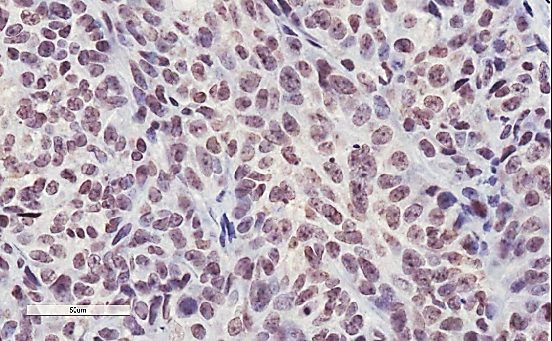 |  | 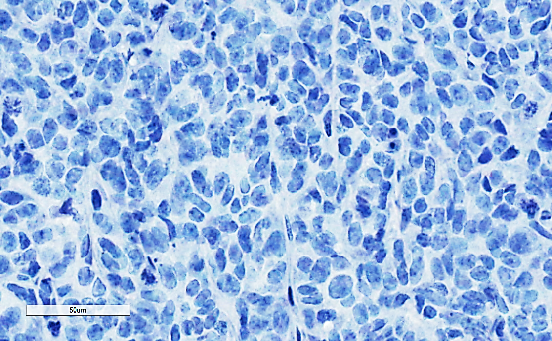 |
| SW480 2W1 | 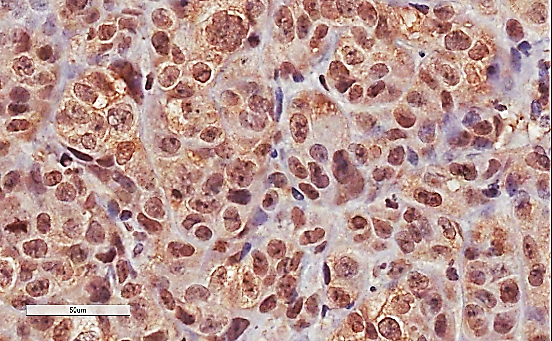 |  | 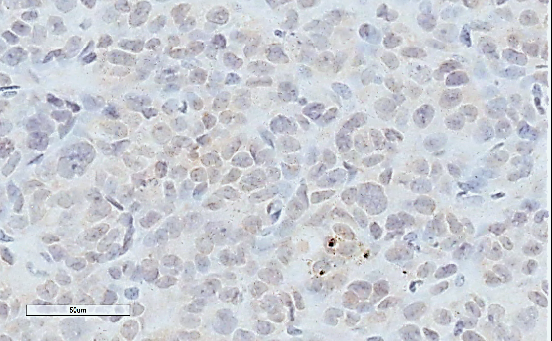 |
| A-253 | 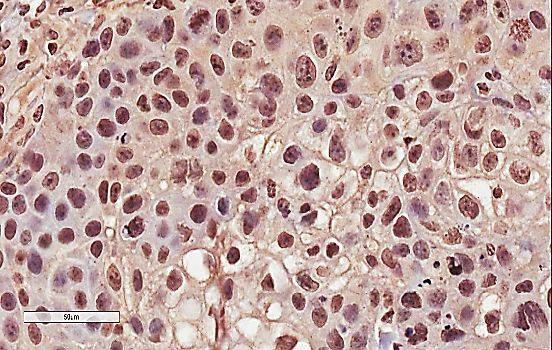 |  | 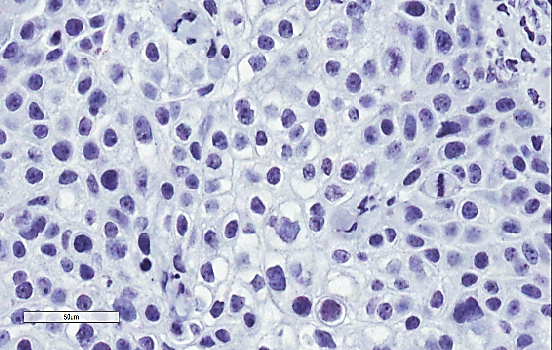 |
| Detroit-562 | 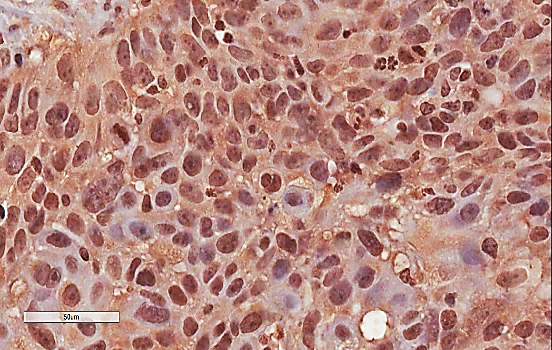 |  | 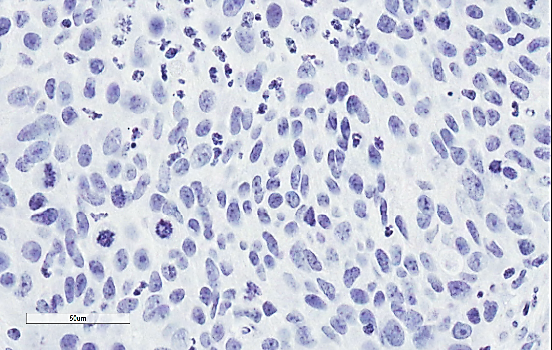 |
| FaDu | 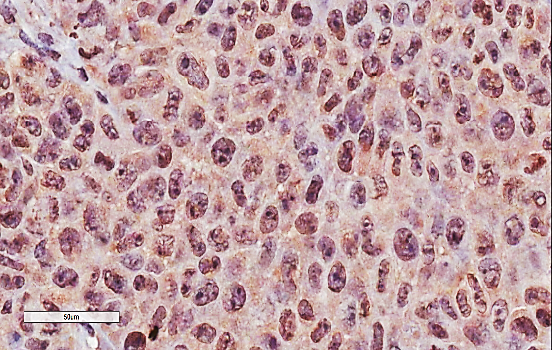 |  | 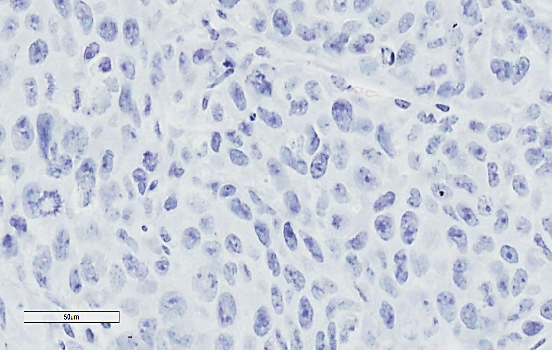 |
| OSC19 | 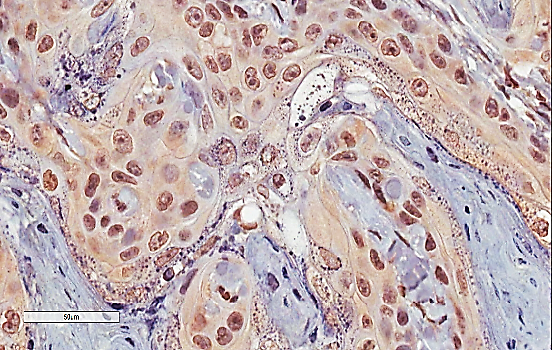 |  | 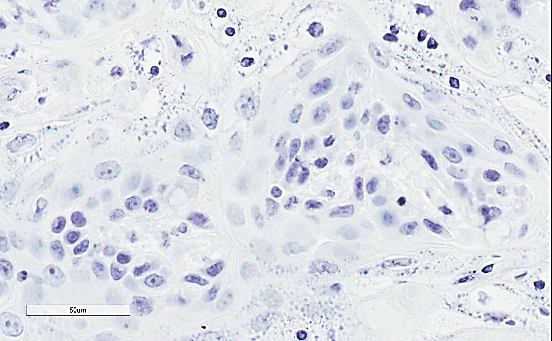 |

**Figure S3C.** Immunohistochemical staining of CYP2W1 in tumour xenografts. Scale bar = 50 μm at 40x magnification.

**Figure S4.** D-Luciferin standard curve and respective equation of linear regression linked to cell-based CYP1A1 P450-Glo™ Assay.


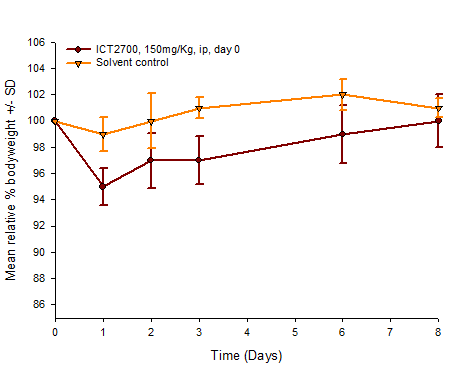
#

**Figure S5.** Bodyweight loss associated with the administration of ICT2700 to FaDu tumours. Mice were treated with a single 150mg/kg dose of ICT2700.


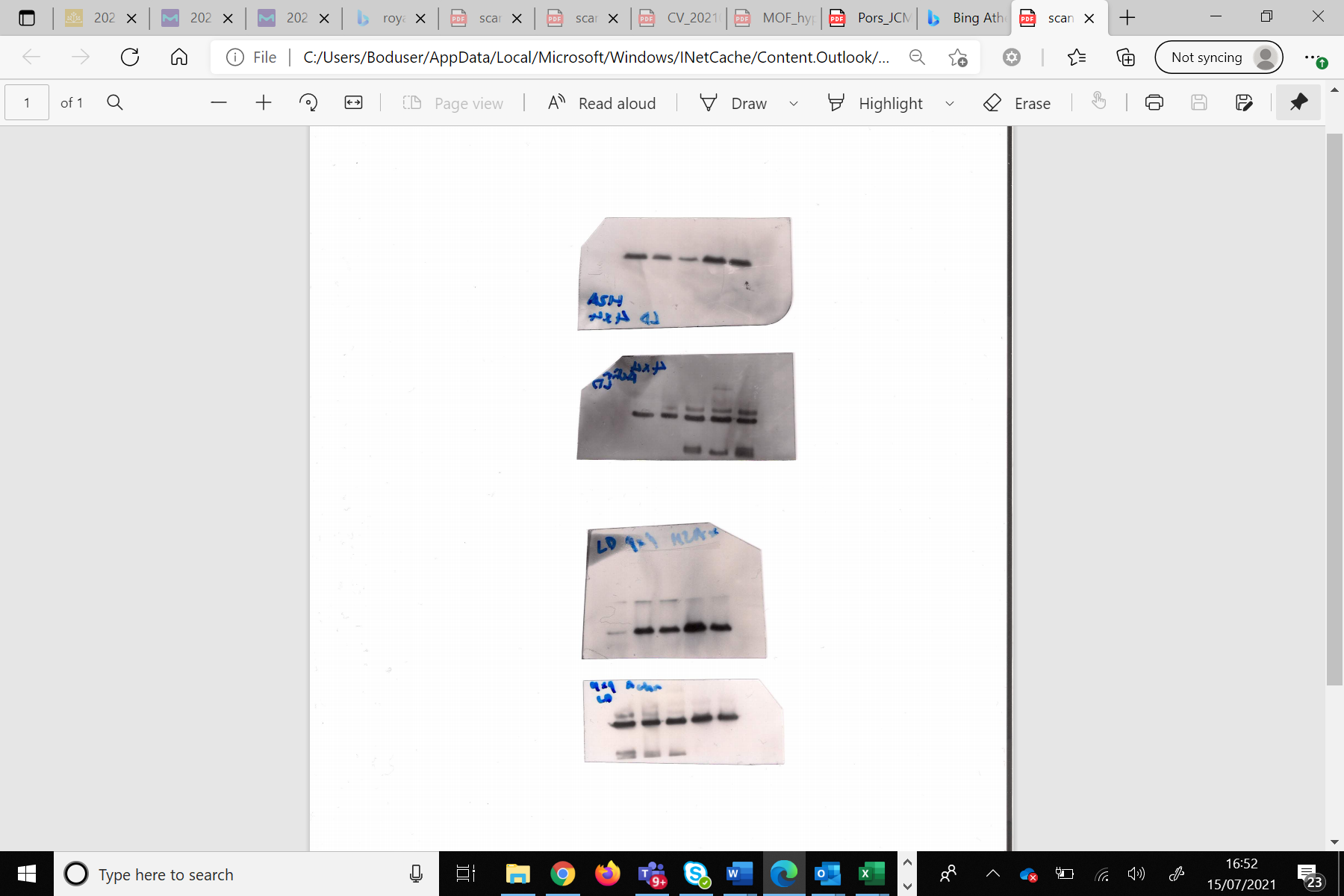


**A**

**B**

**C**

**D**

**Figure S6.** γ-H2AX was used as a marker of DNA double strand damage after 1, 6 and 24hr time points in small (B) and large (C). The top two images (A and B) are full blots from lab book and are presented as Figure 5B in the main manuscript and representing 4x4 tumour and γ-H2AX and β actin, respectively. The bottom two images (C and D) are full blots from lab book and are presented in the main manuscript as Figure 5C representing 9x9 tumours and γ-H2AX and β actin. The top 2 images (4x4) have been flipped horizontally to represent the correct label, as in the manuscript also. These images are raw.
